# Targeting PFKFB3 radiosensitizes cancer cells and suppresses homologous recombination

**DOI:** 10.1038/s41467-018-06287-x

**Published:** 2018-09-24

**Authors:** Nina M. S. Gustafsson, Katarina Färnegårdh, Nadilly Bonagas, Anna Huguet Ninou, Petra Groth, Elisee Wiita, Mattias Jönsson, Kenth Hallberg, Jemina Lehto, Rosa Pennisi, Jessica Martinsson, Carina Norström, Jessica Hollers, Johan Schultz, Martin Andersson, Natalia Markova, Petra Marttila, Baek Kim, Martin Norin, Thomas Olin, Thomas Helleday

**Affiliations:** 1 Science for Life Laboratory, Department of Oncology and Pathology, Karolinska Institutet, 171 21 Stockholm, Sweden; 2 Kancera AB, Karolinska Science Park, 171 48 Solna, Sweden; 3 Drug Discovery and Development Platform, Science for Life Laboratory, Department of Organic Chemistry, Stockholm University, Box 1030, S-171 21 Solna, Sweden; 4 SARomics Biostructures AB, Medicon Village, SE-223 81 Lund, Sweden; 5 Sprint Bioscience, 141 57 Huddinge, Sweden; 6 Department of Sciences, Roma Tre University, 446 00146 Rome, Italy; 7 Department of Pediatrics, Emory University School of Medicine, Atlanta, GA 30322 USA; 8 Malvern Instruments, 752 28 Uppsala, Sweden; 9 Department of Pharmacy, Kyung-Hee University, 02447 Seoul, South Korea; 10 Sheffield Cancer Centre, Department of Oncology and Metabolism, University of Sheffield, S10 2RX Sheffield, UK

## Abstract

The glycolytic PFKFB3 enzyme is widely overexpressed in cancer cells and an emerging anti-cancer target. Here, we identify PFKFB3 as a critical factor in homologous recombination (HR) repair of DNA double-strand breaks. PFKFB3 rapidly relocates into ionizing radiation (IR)-induced nuclear foci in an MRN-ATM-γH2AX-MDC1-dependent manner and co-localizes with DNA damage and HR repair proteins. PFKFB3 relocalization is critical for recruitment of HR proteins, HR activity, and cell survival upon IR. We develop KAN0438757, a small molecule inhibitor that potently targets PFKFB3. Pharmacological PFKFB3 inhibition impairs recruitment of ribonucleotide reductase M2 and deoxynucleotide incorporation upon DNA repair, and reduces dNTP levels. Importantly, KAN0438757 induces radiosensitization in transformed cells while leaving non-transformed cells unaffected. In summary, we identify a key role for PFKFB3 enzymatic activity in HR repair and present KAN0438757, a selective PFKFB3 inhibitor that could potentially be used as a strategy for the treatment of cancer.

## Abstract (web summary)

Targeting the glycolytic PFKFB3 enzyme is being studied as a therapeutic strategy against cancer. Here the authors identify PFKFB3 as being involved in homologous recombination (HR) repair of DNA double strand breaks (DSBs) and present a PFKFB3 inhibitor.

## Introduction

The cellular response to DNA double-strand breaks (DSBs) is orchestrated by the DNA damage response (DDR) where the ataxia-telangiectasia mutated (ATM) kinase plays a central role^1^. ATM rapidly becomes activated by the MRE11/RAD50/NBS1 sensor complex upon ionizing radiation (IR)-induced DSBs^2^. Once activated, ATM phosphorylates the tail of H2AX at Ser139 (γH2AX) on the chromatin flanking the DSB, which attracts binding of the mediator of DNA damage checkpoint protein 1 (MDC1), altogether forming a complex and feedback loop resulting in amplification and stabilization of γH2AX. This serves as a platform for recruitment and accumulation of additional DNA repair factors^3,4^. DSB repair occurs primarily via the error-prone non-homologous end-joining (NHEJ) or with the homologous recombination (HR) pathway in the S and G_2_ phases of the cell cycle, when a sister chromatid is available as a template. The HR process requires DNA end-resection where single-stranded DNA (ssDNA) first is generated via degradation of one of the strands at both sides of the break, a process promoted by BRCA1. The ssDNA overhangs rapidly become coated with the ssDNA binding protein Replication protein A (RPA). Upon initiation of HR, RPA is replaced by the RAD51 recombinase which locates homology in sister chromatids and catalyzes strand invasion and strand pairing^5,6^.

The homodimeric 6-phosphofructo-2-kinase/fructose-2,6-bisphosphatases (PFKFBs) are key regulatory enzymes in the glycolysis^7^. These bifunctional enzymes synthesize and degrade fructose-2,6-bisphosphate (F-2,6-P_2_), which acts as an allosteric activator for the rate-limiting enzyme and committed step in glycolysis, i.e., 6-phophofructo-1-kinase (PFK-1)^8^. In contrast to the PFKFB isoforms 1, 2, and 4, which are constitutively expressed in testes/kidney/heart and liver/muscle, PFKFB3 is an inducible isoform^9^ with increased expression in response to hypoxia, extracellular acidosis, and inflammation. PFKFB3 also stands out with a kinase to bisphosphatase ratio of 740:1, while the other isoforms display a more balanced ratio closer to unity^10^. Consistent with being a transcriptional target of several oncogenic transcription factors (HIF-1α, Akt, PTEN), PKFBF3 protein expression is increased in several cancers seemingly independent of tissue of origin compared to normal matched tissues, making this a recognized target for anti-cancer treatment^11–15^. In addition, a kinase-activating phosphorylation of PFKFB3, resulting in a further elevation of the kinase to bisphosphatase ratio, is more frequently encountered in cancers^16^. High PFKFB3 mRNA expression correlates with poor survival in renal cancer, progression-free, and distant metastatic-free survival in human epidermal growth factor receptor 2 (HER2) positive breast cancer patients^17,18^. Depletion of PFKFB3 by RNA interference in cancer cells delays cell cycle progression and inhibits anchorage-independent cell growth as well as reduces Ras-induced tumor growth in mice^19,20^. Interestingly, a recent study showed potential involvement of cytosolic glycolysis via PFKFB3 in the p53-mediated response to UV damage^21^. However, nuclear PFKFB3 drives cancer cell proliferation without affecting intracellular glycolysis to a measurable extent^22^, suggesting non-canonical functions of PFKFB3 in cancer.

Here, we reveal a role for PFKFB3 in HR repair of DNA DSBs in cancer cells. We demonstrate that PFKFB3 rapidly relocates into IR-induced nuclear foci in an ATM-γH2AX-MDC1-dependent manner and promotes recruitment of HR factors, HR activity, and recovery from IR-induced cell cycle arrest. Through drug discovery efforts, we develop and validate a PFKFB3 inhibitor, KAN0438757, which selectively inhibits proliferation of transformed cells while sparing non-transformed cells. Inhibition of PFKFB3 enzymatic activity by KAN0438757 impairs IR-induced recruitment of ribonucleotide reductase (RNR) M2 and deoxynucleotide incorporation upon DNA repair. Consistent with this, impairment in replication fork progression by KAN0438757 was restored by nucleoside supplementation. In conclusion, we identify a regulatory role for PFKFB3 enzymatic activity in HR repair and our data suggests that PFKFB3 inhibition by KAN0438757 could be an attractive approach to increase sensitivity to therapeutically induced DNA breaks.

## Results

### PFKFB3 is recruited into foci upon ionizing radiation

**Fig. 1:**
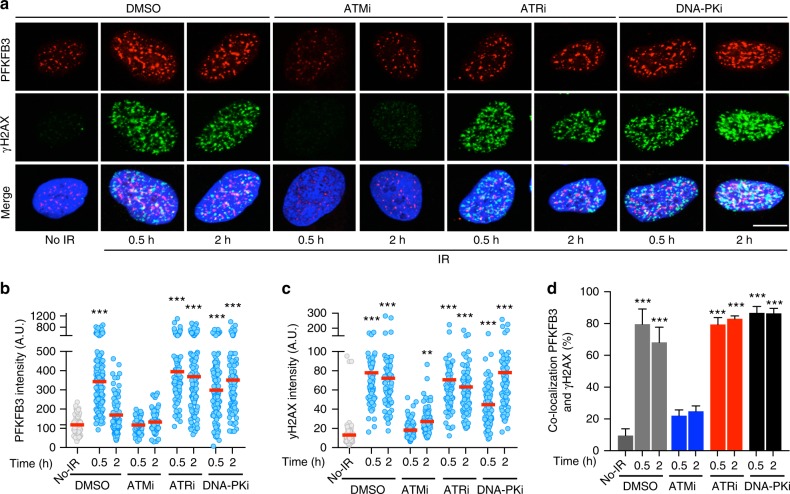
ATM signaling stimulates PFKFB3 recruitment upon DNA damage induction. **a** Confocal analysis of DNA damage, γH2AX, and nuclear localization of PFKFB3 in U2OS cells following treatment with inhibitors (6 h) as indicated, subjected to IR (6 Gy), or left untreated, *n* = 3 independent experiments. Scale bar, 10 μm. **b** Scatter dot plot representing the PFKFB3 nuclear intensity in (**a**) as quantified using CellProfiler, *n* > 100 cells/treatment. ***P < 0.001; one-way ANOVA analysis. **c** Scatter dot plot representing the γH2AX nuclear intensity in (**a**) as quantified using CellProfiler, *n* > 100 cells/treatment. **P < 0.01, ***P < 0.001; one-way ANOVA analysis. **d** Bars representing the percentage of PFKFB3 and γH2AX foci that co-localize in (**a**) as quantified using CellProfiler, *n* > 100 cells/treatment. Data are displayed as means ± SEM, ***P < 0.001, one-way ANOVA was used to calculate statistical significance

In an analysis of publically available microarray data sets, we identified the PFKFB3 mRNA to be upregulated in radiotherapy resistant patients both before and after radiotherapy compared to radiosensitive patients (Supplementary Figure 1). These resistant patients are marked by increased ability to repair IR-induced DNA breaks^23^. This together with identifying PFKFB3 in genome-wide siRNA screens aiming to detect DDR factors^24,25^, prompted our interest in investigating a previously unknown role for PFKFB3 in HR repair. To investigate a potential role for PFKFB3 in DSB repair, we determined the localization of PFKFB3 using in situ cell fractionation^26^ following induction of DSBs by IR in U2OS and BJ RAS (hTERT immortalized fibroblasts transformed with SV40 and RAS) cells. We observed a rapid increase in focal accumulation of PFKFB3 in the nuclei that reached maximum levels at 0.5 h post IR coinciding with the induction of phosphorylated H2AX (γH2AX) (Fig. 1a–c, Supplementary Figure 3a, b). The IR-induced increase in PFKFB3 was dynamic and decreased at 2 h, while the γH2AX levels remained high, thus PFKFB3 is recruited early in the DDR (Fig. 1a, b). The increase of PFKFB3 and γH2AX upon IR also coincided with them co-localizing in foci, notably the co-localization remained high at 2 h post-IR (Fig. 1d).

To gain insight into the mechanism behind this, rapid accumulation of PFKFB3, U2OS, and BJ RAS cells were treated with inhibitors against the most upstream DDR kinases ATM, ATM- and Rad3-related (ATR) as well as the DNA-dependent protein kinase (DNA-PK) prior to IR (Supplementary Figure 2a). The IR-induced focal accumulation of PFKFB3 and γH2AX, as well as co-localization between the proteins, was blocked upon ATM inhibition, the major transducer in the IR-induced DDR (Fig. 1a–c, Supplementary Figure 3a, b). Neither inhibition of ATR nor DNA-PK affected the rapid recruitment of PFKFB3 or its co-localization with γH2AX upon IR and resulted in retention of PFKFB3 at 2 h post-IR (Fig. 1a–c, Supplementary Figure 3a, b). Consistent with ATM being the major transducer in the IR-induced DDR response^27^, ATR and DNA-PK inhibition did not impair the γH2AX recruitment upon IR as compared to DMSO treatment (Fig. 1a, c, Supplementary Figure 3a, b). However, both ATR and DNA-PK are important for functional DDR^27^, suggesting that PFKFB3 is retained in nuclear foci upon impaired DDR signaling downstream of ATM and γH2AX. Consistent with activation of ATM upon DNA damage being under the dependence of the MRN complex^28^, inhibition of the MRN complex by Mirin^29^ blocked the IR-induced recruitment of PFKFB3 (Supplementary Figure 3c).

**Fig. 2:**
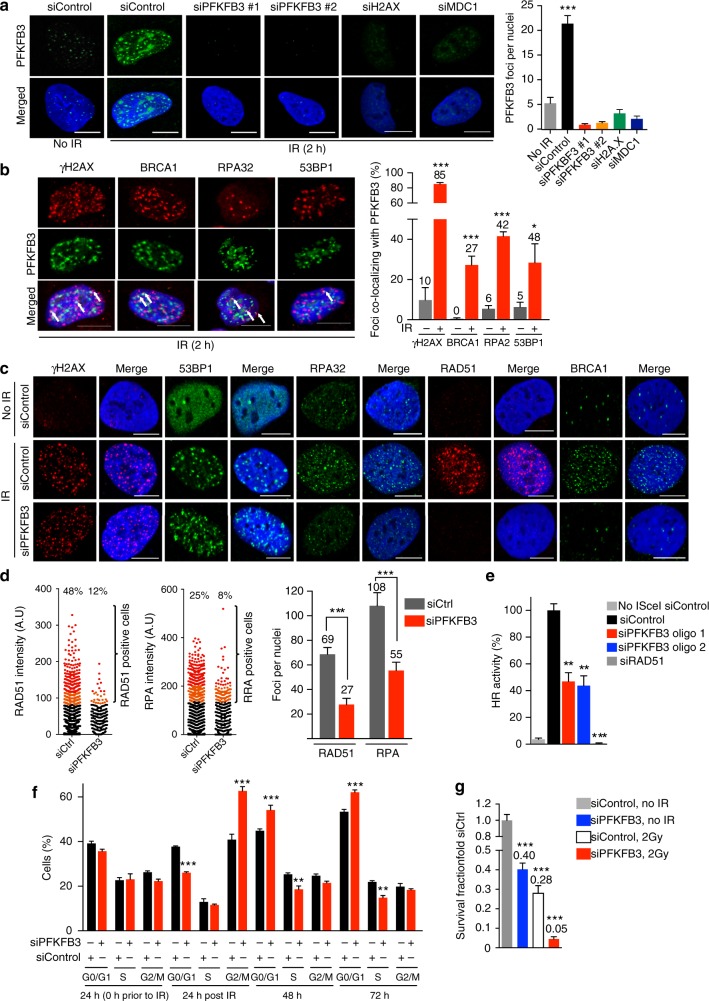
PFKFB3 promotes homologous recombination repair. **a** Confocal analysis of nuclear localization of PFKFB3 in U2OS cells following treatment with siRNAs (24 h) as indicated, subjected to IR (6 Gy, 2 h recovery), or left untreated, *n* = 3 independent experiments. Scale bar, 10 μm. To the right, bar chart showing PFKFB3 foci as quantified using CellProfiler, *n* > 100 cells/treatment. Data are displayed as means ± SEM. ***P < 0.001; one-way ANOVA analysis. **b** Confocal images of co-localization between PFKFB3 and indicated proteins in U2OS cells upon IR (6 Gy, 2 h recovery). White arrows indicate co-localized foci, *n* = 2 independent experiments. Scale bar, 10 μm. To the right, bar graph representing the percentage of PFKFB3 foci that co-localized with indicated proteins as quantified using CellProfiler. *n* > 100 cells/condition. Data displayed as means ± SEM. ***P < 0.001, *P < 0.05; Student’s *t*-test. **c** U2OS cells were treated with indicated siRNAs for 24 h, subjected to IR at 6 Gy (2 h recovery) and immunostained for indicated proteins, *n* = 3 independent experiments. Scale bar, 10 μm. **d** Scatter dot plot representing the RAD51 or RPA32 nuclear intensity in (**c**) as quantified using CellProfiler. To the right, bars showing RAD51 or RPA foci per nuclei in (**c**). *n* > 500 cells/treatment. Data displayed as means ± SEM. ***P < 0.001; Student’s *t*-test. **e** HR activity after treatment of U2OS DR-GFP cells with indicated siRNAs as assessed by FACS analysis, whereby the siControl cells are set as reference cells (100% activity). Data are displayed as means ± SEM, *n* = 3. **P < 0.01, ***P < 0.001; one-way ANOVA analysis. **f** DNA histograms of U2OS cells treated with indicated siRNAs for 24 h then subjected to IR at 2 Gy (24 h recovery) or left untreated for 48 and 72 h. Cells were fixed and DNA content was assessed using propidium iodide staining and flow cytometry. Data are displayed as means ± SEM, *n* = 3. **P < 0.01, ***P < 0.001; one-way ANOVA analysis. **g** Colony formation of U2OS cells 10 days after treatment with 10 nM siControl or 10 nM siPFKFB3 in combination with 2 Gy or left untreated, was assessed by staining with 4% methylene blue in methanol and counting colonies. Data are displayed as means fold over siControl no IR ± SEM, *n* > 5. ***P < 0.001; one-way ANOVA analysis

To elucidate the signaling pathways downstream of ATM that modulate PFKFB3 recruitment we next inhibited γH2AX and MDC1 by siRNA (Supplementary Figure 2b, c). RNAi-mediated silencing of H2AX and MDC1 effectively reduced the recruitment of PFKFB3 into IR-induced foci (Fig. 2a). The PFKFB3 foci were also abolished with two different siRNAs targeting PFKFB3 indicating specific staining (Fig. 2a, Supplementary Figure 2d). Thus, we conclude that upon IR, the MRN complex, ATM, γH2AX, and MDC1 stimulate re-localization of PFKFB3 into nuclear foci.

### PFKFB3 regulates homologous recombination repair

To examine the role of PFKFB3 in DNA repair in greater detail, we assessed co-localization between PFKFB3 foci and foci of ATM-dependent DNA damage and repair factors upon induction of DSBs by IR in U2OS and BJ RAS cells. PFKFB3 foci co-localized with IR-induced foci of γH2AX, 53BP1, RPA, and BRCA1 (Fig. 2b, Supplementary Figure 4). To investigate whether the recruitment of these factors is dependent on PFKFB3, we induced DSBs by IR in PFKFB3 siRNA transfected cells. PFKFB3 knockdown abolished recruitment of the HR repair factors RPA32 and BRCA1 into foci upon IR, while focal accumulation of the upstream DNA damage markers 53BP1 and γH2AX was unaffected (Fig. 2c, d). Notably, also recruitment of RAD51 was blocked upon PFKFB3 knockdown (Fig. 2c, d). As RPA32, BRCA1, and RAD51 are essential for functional HR repair, we next evaluated the importance of PFKFB3 in HR efficiency employing the DR-GFP assay in U2OS cells^30^. Importantly, HR repair activity was decreased down to 40% upon knockdown of PFKFB3 with two different siRNAs (Fig. 2e). To confirm that the block in HR repair activity upon PKFBF3 silencing was not a secondary effect due to a potential reduction of cells in the S-G2/M phases, we performed cell cycle analysis at the time for the DR-GFP assay (72 h siRNA treatment). The PFKFB3 knockdown population displayed a minor decrease of cells of approximately 7% in the S phase, however, this modest drop is unlikely to account for a 60% decrease in HR activity (Fig. 2f).

IR delays cell cycle progression via ATM, which also signals for DNA repair prior to entry into mitosis^3^. Impaired HR will result in delayed or non-functional DNA repair. Thus, we next investigated potential effects on the cell cycle distribution upon PFKFB3 depletion in combination with IR. While untreated control cells displayed a normal G2/M population upon silencing of PFKFB3, the IR-treated cells showed a delayed G2/M phase progression compared to control (Fig. 2f). To determine the long-term effects on cancer cell survival upon PFKFB3 depletion in combination with IR, clonogenic survival assays were performed. Consistent with PFKFB3 supporting cancer cell proliferation, PFKFB3 silencing on its own reduced clonogenic survival (Fig. 2g). Importantly, PFKFB3 silencing significantly enhanced radiosensitivity with approximately 6-fold as compared siControl treatment with IR (Survival Fraction 0.05 versus 0.28) (Fig. 2g). Thus, we conclude that upon IR, PFKFB3 is required for recruitment of RPA and RAD51, functional HR repair of DSBs, cell cycle progression, and long-term cell survival.

### Development of PFKFB3 inhibitor

To further evaluate the role of PFKFB3 enzymatic activity in supporting HR, we treated U2OS cells with the previously described PFKFB3 inhibitor 3-(3-pyridinyl)-1-(4-pyridinyl)-2-propen-1-one (3-PO)^31^ in combination with IR. However, IR-induced recruitment of RPA32 and RAD51 to DNA breaks was unaffected in the presence of 3-PO (Supplementary Figure 5a, b), inconsistent with the PFKFB3 siRNA data (Fig. 2c). We also used the DR-GFP reporter systems to evaluate the HR repair activity. However, treatment of U2OS cells with 3-PO did not decrease HR activity (Supplementary Figure 5c), again inconsistent with the results from PFKFB3 siRNA experiments (Fig. 2e). To further understand the utility of 3-PO as a PFKFB3 inhibitor, its effect on human recombinant PFKFB3 enzyme as well as production of fructose-2,6-bisphosphatase in cells were studied. In line with previous publications^32,33^, we could not achieve an IC_50_ value for the 3-PO compound in the PFKFB3 activity assays (Supplementary Figure 5d). The apparent lack of PFKFB3 inhibition in our hands, in combination with the lack of an available crystal structure confirming binding of 3-PO to PFKFB3, prompted us to search for alternative inhibitors.

**Fig. 3:**
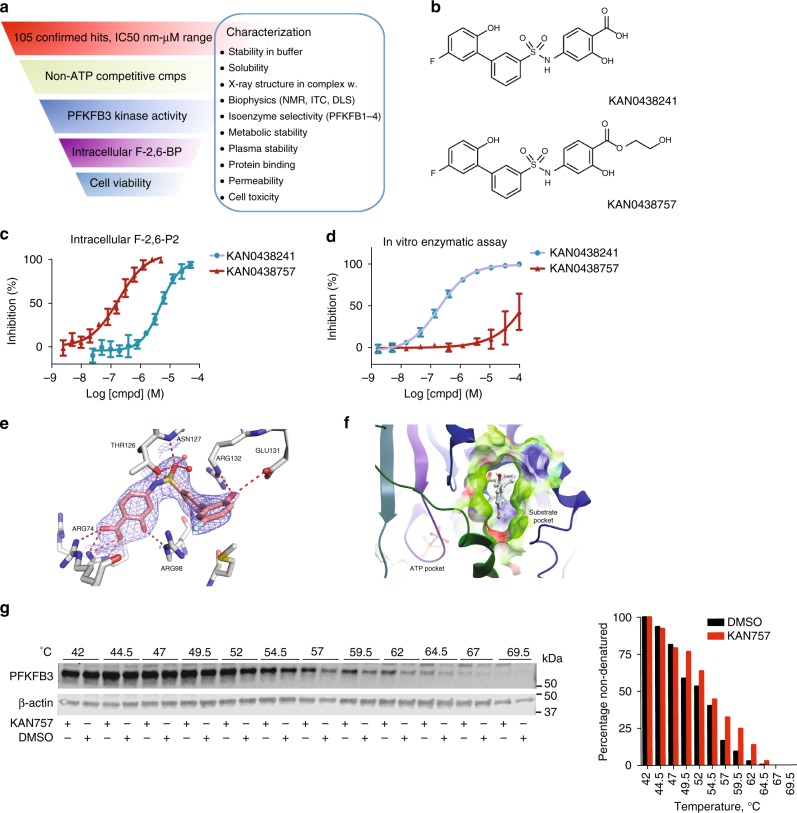
Development of PFKFB3 inhibitor. **a** Illustrative description of the drug discovery process towards identification of KAN0438757 as a PFKFB3 inhibitor. Following measurements of inhibition of human recombinant enzyme, the inhibitory effects on PFKFB3 in cells were demonstrated by intracellular measurements of F-2,6-BP levels by the van Schaftingen assay, thereafter effects on cell viability in different cancer cells were assessed. In parallel, biophysical studies were used to validate mechanism-of-action, selectivity towards related isoenzymes was tested to ascertain a promising selectivity profile, and different ADME assays were carried out to identify a molecule with promising properties. **b** Chemical structures of the PFKFB3 inhibitors KAN0438241 and the pro-drug KAN0438757. **c** Comparison of the acid KAN0438241 and its pro-drug KAN0438757 on inhibition of F-2,6-P2 level using the van Shaftingen assay in cells, *n* *=* 4. **d** Comparison of the acid KAN0438241 and its pro-drug KAN0438757 on inhibition of human recombinant PFKFB3 kinase activity. Activity was quantified based on the production of ADP and F-2,6-P2 from ATP and F6P, *n* *=* 5. **e** Stick model of KAN0438241 bound to human PFKFB3. The compound is bound to the fructose-6-phosphate substrate pocket. The stick model is colored according to atom type: oxygen in red, nitrogen in blue, sulfur in yellow, and carbon in white (protein) or pink (KAN0438241). Polar contacts between the compound and protein residues are shown as red dotted lines. The final 2Fo-Fc electron density map for the compound, contoured at 1 s, is shown as blue mesh. The image was prepared using PyMOL^1^. **f** X-ray structure of human recombinant PFKFB3 in complex with KAN0438241. Crystal structure of KAN04438241 (stickmodel, pdb code 6ETJ) in the substrate pocket of the catalytic domain of PFKFB3. The molecular surface of the pocket is depicted color coded according to the calculated electrostatic charge (positive in blue, negative in red, and neutral in green). The ATP binding site is indicated in the low left part of the picture. The image was generated using the ICM-pro software from Molsoft LLC (www.molsoft.com). **g** Target engagement and thermal stabilization of PFKFB3 by 10 µM KAN757 (KAN0438757) as compared to DMSO in U2OS cells, assessed by CETSA. Band intensities were quantified using ImageJ, PFKBF3 levels were normalized against β-actin. To the right, bar graph representing the percentage of non-denatured PFKFB3 relative to DMSO at 42 °C, shown is a representative experiment of *n* = 2

In the search for inhibitors of the enzymatic activity of PFKFB3, we screened 50,000 diverse compounds (Fig. 3a, Supplementary Fig. 6a). Representative compounds from hit series and singletons were tested for binding to PFKFB3 using Saturation Transfer Difference NMR to confirm reversible binding. Competition experiments with ATP were used to identify ATP-competitive and non-ATP-competitive binders (Supplementary Figure 6b). This effort lead to the identification and further development of a series of phenylsulfonamido salicylic acids that rendered sub-µM inhibitors of PFKFB3. These non-ATP-competitive ligands were selected for further optimization to avoid cross activity with other kinases (Fig. 3a, Supplementary Figure 6b). A representative of this compound series is illustrated by KAN0438241 in Fig. 3b, which demonstrated selectivity for the kinase activity of PFKFB3 versus the other isoforms PFKFB1, PFKFB2, and PFKFB4 (Supplementary Figure 6c). Significant cross-reactivity is only observed for PFKFB4, although the selectivity window is about 20-fold with IC_50_ values of 0.19 and 3.6 µM, respectively (Supplementary Figure 6c). The sub-µM potency for PFKFB3 was also confirmed using isothermal titration calorimetry as illustrated in Figure S6D and co-crystal structures demonstrated binding in the active site, mimicking several of the interactions made by the endogenous substrate fructose-6-phosphate (F6P) (Fig. 3e).

Further characterization of KAN0438241 and its ester KAN0438757 (Fig. 3b), which was designed to increase cell permeability and efficient intracellular hydrolysis to release the active compound, involved examination of their impact on the proximal biomarker F-2,6-P_2_ in several different cell lines^34^. Whereas the salicylate was only weakly active with low µM potency in the cellular setting (Fig. 3c), the ester demonstrated significant suppression of intracellular F-2,6-P_2_ levels with sub-µM IC_50_ values in cell lines representing pancreatic, gastric, and colon cancer (Fig. 3c, Supplementary Figure 6e). In the same cell lines, KAN0438757 was also demonstrated to significantly reduce cell viability following prolonged incubation times (Figure S6F). Consistent with PFKFB3 being overexpressed in cancer, the viability of peripheral blood mononuclear cells (PBMCs) was not affected upon inhibition of PFKFB3 by KAN0438757 (Supplementary Figure 6f).

The effect on intracellular F-2,6-P_2_ levels is in contrast to what we observed in the in vitro enzymatic assay for PFKFB3, where the ester is largely inactive (Fig. 3d). Given the binding pose, which involves important interactions for the carboxylate (Fig. 3e, f), the lack of activity for the ester in a non-cellular setting is not surprising. Using the cellular thermal shift assay (CETSA)^35,36^, we could also confirm that KAN0438757 demonstrated intracellular target engagement for PFKFB3 in the same concentration range as that observed using the Van Schaftingen assay (Fig. 3g). The target engagement by KAN0438757 persisted at 24 and 72 h of treatment (Supplementary Figure 7a–d), with the later time-point corresponding to the viability measurement, indicating PFKFB3 inhibition and compound stability during the entire time-course of the viability assay. To further validate KAN0438241 as a useful chemical tool for probing cellular PFKFB3 activity, we examined the kinome-wide selectivity profile of KAN0438757 and its active metabolite KAN0438241 with DiscoveRx’s KINOMEscan technology^37^. Both compounds displayed high selectivity (SScore(35) = 0) at 2 µM. The results demonstrated that no significant binding could be detected in any of the 97 targets in the diverse scanEDGE^sm^ set of kinases (Supplementary Figure 6g and Supplementary Data 1).

### DNA repair is dependent on PFKFB3 kinase activity

**Fig. 4:**
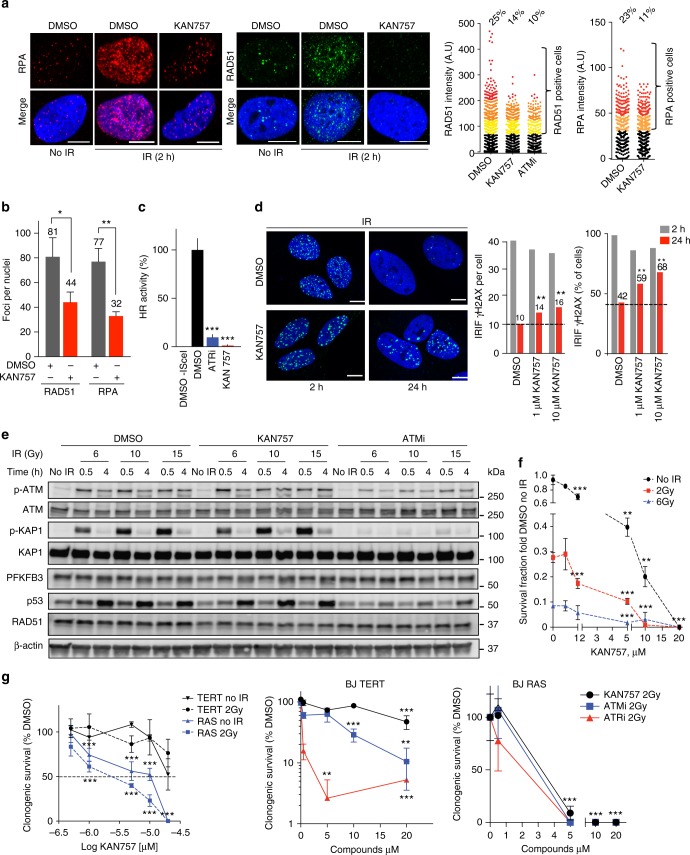
PFKFB3 kinase activity is needed for effective DNA repair. **a** Confocal analysis of IR-induced foci of RAD51 or RPA32 in U2OS cells following treatment with DMSO or 10 μM KAN757 (6 h), subjected to IR (6 Gy, 2 h recovery), or left untreated. Shown is a representative experiment of *n* = 3. Scale bar, 10 μm. To the right, scatter dot plot representing the intensity of the fluorescent levels of RAD51 or RPA32 as assessed by quantifying nuclear intensity using CellProfiler, *n* > 500 cells/treatment. **b** Bar chart showing the percentage of foci per nuclei in (**a**) as quantified using CellProfiler. Data are displayed as means ± SEM, **P < 0.01, *P < 0.05; Student’s *t*-test. **c** HR activity after treatment of U2OS DR-GFP cells with indicated inhibitors as assessed by FACS analysis. DMSO treated cells are taken as reference cells (100% activity). Data are displayed as means ± SEM, *n* = 3. ***P < 0.001; Student’s *t*-test. **d** DNA damage levels were assessed using γH2AX staining, U2OS cells were treated with DMSO or KAN0438757 for 6 h, subjected to IR (6 Gy, 2 h, and 24 h recovery), and immunostained for γH2AX. Scale bar, 10 μm. Right panel, quantification of average γH2AX IRIF per cell and percentage of γH2AX positive cells (>10 γH2AX foci per cell) using CellProfiler. Data are displayed as means, *n* > 500 cells/treatment. **P < 0.01; one-way ANOVA analysis. **e** U2OS cells were treated with DMSO, 10 μM of KAN757 or 10 μM of KU55933 (ATM inhibitor) for 24 h, exposed to IR (6, 10, and 15 Gy) and harvested at the indicated time points. Whole cell extracts were prepared and protein levels were analyzed by western blot using indicated antibodies, β-actin was used as loading control. Shown is a representative experiment of *n* = 3. **f** Colony formation of U2OS cells upon PFKFB3 inhibition. U2OS cells were treated with DMSO or 10 μM KAN757 for 3 days, then exposed to indicated doses of IR or left untreated, inhibitors were washed out and 5 days later colonies were stained with 4% methylene blue-MeOH and counted. Data are displayed as means ± SEM, *n* = 3. **P < 0.01, ***P < 0.001; one-way ANOVA analysis. **g** Colony formation upon PFKFB3 inhibition in transformed and non-transformed cells. BJ TERT or RAS cells were treated with vehicle (DMSO) or indicated concentrations of inhibitors for 3 days, exposed to IR (2 Gy), or left untreated. Inhibitors were washed out 24 h post IR and 4 days (RAS) or 7 days (TERT) later colonies were stained with 4% methylene blue-MeOH and counted. Data are displayed as means ± SEM, *n* > 3. **P < 0.01, ***P < 0.001; one-way ANOVA analysis. KAN757 = KAN0438757

To investigate if the impaired HR upon PFKFB3 silencing is dependent on the kinase activity of PFKFB3, we examined the IR-induced recruitment of RPA32 and RAD51 into repair foci in KAN0438757-treated U2OS cells. The recruitment of both HR factors was disrupted upon inhibition of PFKFB3 (Fig. 4a, b), consistent with the results observed upon inhibition of PFKFB3 expression using siRNA knockdown (Fig. 2d). Consistent with PFKFB3 being downstream of the MRN complex, treatment with KAN0438757 prior to IR did not affect the IR-induced Mre11 or Nbs1 foci formation in comparison to treatment with Mirin that completely blocked induction of both the components of the MRN complex (Supplementary Figure 8a, b). Due to the decreased recruitment of RPA and RAD51, the DR-GFP reporter system was next used to investigate the impact of KAN0438757 on HR repair activity. Treatment of U2OS cells with KAN0438757 decreased HR activity down to 10% as compared to vehicle-treated cells and notably even more so than inhibition of ATR, a key component in HR (Fig. 4c). To confirm that the block in HR repair activity upon PKFBF3 inhibition was not a secondary effect due to a potential reduction of cells in the S-G2/M phases, we performed cell cycle analysis at the time for the DR-GFP assay. Similar to PFKFB3 knockdown, the KAN0438757 population displayed a modest decrease of cells in the S phase (Supplementary Figure 8c). Notably, treatment with the 3-PO compound resulted in a similar cell cycle profile as KAN0438757 and siPFKFB3 (Supplementary Figure 8c), but without affecting HR activity (Supplementary Figure 5c). Thus, the small effect on the cell cycle distribution upon PFKFB3 knockdown or inhibition is not sufficient to alter the HR activity in the DR-GFP assay.

We hypothesized that disrupted HR activity upon PFKFB3 inhibition in combination with IR would result in unrepaired DSBs and thereby sustained high levels of DSBs. Thus we quantified the focal accumulation of γH2AX, a sensitive marker of DSBs^38^, at 2 and 24 h post-IR in KAN0438757-treated cells. PFKFB3 inhibited cells had significantly higher levels of γH2AX foci, as well as the percentage of γH2AX positive cells, compared to vehicle at 24 h post-IR (Fig. 4d), indicative of reduced repair of the IR-induced DNA damage. Consistent with the impaired HR activity and sustained high levels of γH2AX at 24 h post-IR in PFKFB3 inhibitor-treated cells, we observed an increased accumulation of cells in the G2/M phase upon IR in combination with KAN0438757 treatment at 24 h post-IR, a phenotype not seen with 3-PO (Supplementary Figure 8c).

We next assessed how KAN0438757 affects DDR signaling following IR and in comparison to ATM inhibition. We observed that PFKFB3 protein levels are not affected by KAN0438757 or ATM inhibitor treatments nor do we detect any protein modifications on PFKFB3 upon IR (Fig. 4e). Thus, the IR-induced accumulation of PFKFB3 (Figs. 1 and 2) is likely due to recruitment and not due to a global increase in PFKFB3 protein levels. Consistent with PFKFB3 being downstream of MRN, ATM, γH2AX, and MDC1 (Fig. 1), KAN0438757 did not affect IR-induced phosphorylation of ATM, H2AX, or KAP-1 (Fig. 4e). RAD51 expression levels were assessed in order to confirm that the KAN0438757-dependent decrease in RAD51 recruitment in Fig. 4a was not due to decreased RAD51 protein levels (Fig. 4e).

We next performed clonogenic survival assays, in which U2OS cells pre-treated with KAN0438757 prior to IR displayed a dose-dependent radiosensitization to form colonies 10 days post treatments (Fig. 4f). To determine if the decreased clonogenic survival upon inhibition was due to lack of recruitment of RAD51, we overexpressed RAD51 prior to treatment with KAN0438757 and IR. Notably, RAD51 overexpression rescued the clonogenic survival at the IC_50_ concentration of KAN0438757 in the assay (Supplementary Figure 8d), suggesting a loss of HR by KAN0438757 contributes to the toxicity.

Since PFKFB3 protein levels are increased in cancer versus normal matched tissues^13^, we next compared the effect of KAN0438757 in isogenic immortalized and transformed cells. BJ TERT (hTERT immortalized normal fibroblasts) and BJ RAS were treated with KAN0438757 or vehicle for 24 h and then subjected to IR or left untreated. The inhibitors were washed out 72 h later and colonies were counted 4 days (BJ RAS) or 7 days (BJ TERT) later. The BJ RAS cells displayed decreased clonogenic survival, in a dose-dependent manner, upon KAN0438757 treatment at concentrations that only marginally affected BJ TERT both in conditions with and without IR (Fig. 4g, left graph). This indicates tolerability to KAN0438757 by normal cells at concentrations enhancing radiosensitivity and disrupting survival in cancer cells. In comparison, inhibition of ATM and ATR in combination with IR resulted in a 10% or less survival of the non-transformed BJ TERT cells, while being equally effective as KAN0438757 at inhibiting survival of the transformed BJ RAS cells (Fig. 4g, middle and right graphs). These data suggest that PFKFB3 inhibition induces DNA repair deficiency specifically in transformed cells, while being expendable to non-transformed cells in contrast to ATM and ATR. Altogether, our data show that PFKFB3 kinase activity is involved in the recruitment of DNA repair factors upon IR, successful DNA repair, recovery from IR-induced cell cycle arrest, and long-term survival of transformed cells.

### PFKFB3 promotes nucleotide incorporation during DNA repair

**Fig. 5:**
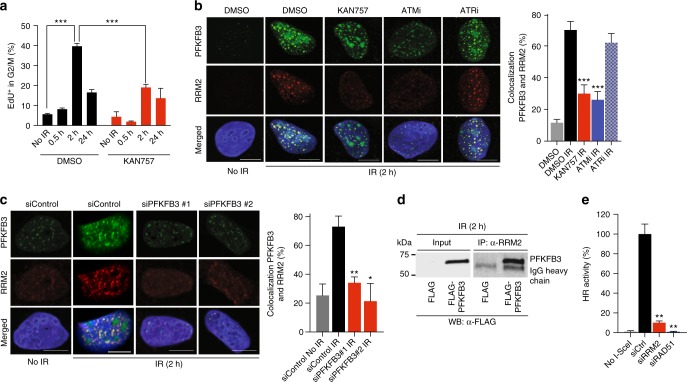
PFKFB3 kinase activity supports DNA repair synthesis. **a** U2OS cells were treated with DMSO or 10 μM KAN757 (6 h), exposed to IR (6 Gy, 0.5 h, 2 h, and 24 h recovery), or left untreated. At each time-point, cells were pulsed 30 min with 10 µM EdU, harvested, and fixed. DNA was stained with Hoechst and EdU detected by fluorophore conjugation for flow cytometry analysis. Bars indicate the number of EdU-positive cells in the G2/M phase. Data are displayed as means ± SEM, *n* = 3. ***P < 0.001; one-way ANOVA analysis. KAN757 = KAN0438757. **b** Representative images of confocal analysis of IR-induced foci of PFKFB3 and RRM2 in U2OS cells following treatments (6 h) as indicated, subjected to IR (6 Gy, 2 h recovery), or left untreated. Bar chart on the right shows the percentage of cells with PFKFB3 foci co-localizing with RRM2 foci (>10 colocalizing foci/cell), >100 cells/condition. Data are displayed as means ± SEM, *n* = 2. ***P < 0.001; one-way ANOVA analysis. KAN757 = KAN0438757. **c** Confocal analysis of RRM2 and PFKFB3 recruitment upon IR (6 Gy, 2 h recovery) in U2OS cells treated with indicated siRNA for 24 h or left untreated. To the right, bar chart showing quantification of cells with PFKFB3 foci co-localizing with RRM2 foci (>10 colocalizing foci/cell), >100 cells/condition. Data are displayed as means ± SEM, *n* = 2. **P < 0.01, *P < 0.05; one-way ANOVA analysis. **d** FLAG-PFKFB3 or FLAG transfected U2OS cells were exposed to IR (6 Gy) or left untreated. At 2 h post-IR cells were subjected to immunoprecipitations followed by immunoblot with FLAG antibody. Shown is a representative experiment of *n* = 3. **e** HR activity after treatment of U2OS DR-GFP cells with indicated siRNAs as assessed by FACS analysis, whereby the siControl cells are set as reference cells (100% activity). Data are displayed as mean ± SEM, *n* = 3. **P < 0.01; one-way ANOVA analysis

Given the role of PFKFB3 in the generation of deoxyribonucleotide triphosphates (dNTPs), we hypothesized a role of PFKFB3 in HR-dependent repair synthesis. To test this directly, the kinetics of incorporation of deoxynucleotides during DNA repair was investigated by assessing thymidine analogue 5-ethynyl-2′-deoxyuridine (EdU) incorporation into DNA in the G2/M phase upon IR. Notably, treatment with KAN0438757 prior to IR impaired the IR-induced increase in the EdU-positive cell population in the G2/M phase (Fig. 5a), suggesting the involvement of the kinase activity of PFKFB3 in deoxynucleotide incorporation during HR-dependent repair synthesis. In this analysis, we excluded cells in the S phase to disregard contributions from EdU incorporation during replication rather than as a result of DNA repair. The possible reasons why PFKFB3 inhibited cells fail to incorporate deoxynucleotides during DNA repair could be either that PFKFB3 supports localized dNTP supply via its role in glycolysis or that it recruits a factor needed for dNTP production. Given the established role of PFKFB3 in supporting glycolysis including its diversion towards the synthesis of the nucleotide precursor PRPP, it is easy to envision a potential role of the IR-induced recruitment of PFKFB3 in localized dNTP production via its role in glycolysis. There is an obvious issue with this hypothesis, as glycolysis normally takes place in the cytoplasm where the F-2,6-P_2_ activator is well established as a positive allosteric regulator of PFK-1, suggesting this is unlikely. The RRM2 subunit of the RNR localizes to the nuclei in response to IR to ensure local dNTP production for repair DNA synthesis^39,40^, thus we hypothesized that PFKFB3 may support the supply of dNTPs for DSB repair via recruitment of RRM2. To test this, we assayed to what extent modulation of PFKFB3 activity impacts RRM2 recruitment upon IR. Both PFKFB3 and RRM2 were recruited into repair foci in an ATM-, but not ATR-, dependent manner upon IR (Fig. 5b). Importantly, the enzymes co-localized and this was disrupted upon pre-treatment with KAN0438757 or an ATM inhibitor prior to IR (Fig. 5b). Knockdown of PFKFB3 with two different oligos also abolished the IR-induced recruitment of RRM2 as well as the IR-induced co-localization between PFKFB3 and RRM2 (Fig. 5c). To investigate a possible physical interaction between PFKFB3 and RRM2, we next performed co-immunoprecipitation experiments. Consistent with our observation that PFKFB3 and RRM2 co-localize upon IR, FLAG-PFKFB3 was brought down by RRM2 antibodies at 2 h post-IR (Fig. 5d), while we could not detect any interaction between FLAG-PFKFB3 and RRM2 in non-IR conditions (Supplementary Figure 9).

To evaluate the contribution of RRM2 to functional HR, we measured HR activity in the DR-GFP assay upon treatment with RRM2 siRNA. Knockdown of RRM2 reduced the HR activity to approximately 10% (Fig. 5e, Supplementary Figure 2g), similar to what was observed upon PFKFB3 inhibition. Thus, the block in HR repair upon PFKFB3 inhibition could be due to insufficient nucleotide availability as a result of impaired recruitment of RRM2, suggesting the involvement of PFKFB3 activity in nucleotide incorporation during repair synthesis.

### PFKFB3 supports dNTP supply in DNA synthesis

**Fig. 6:**
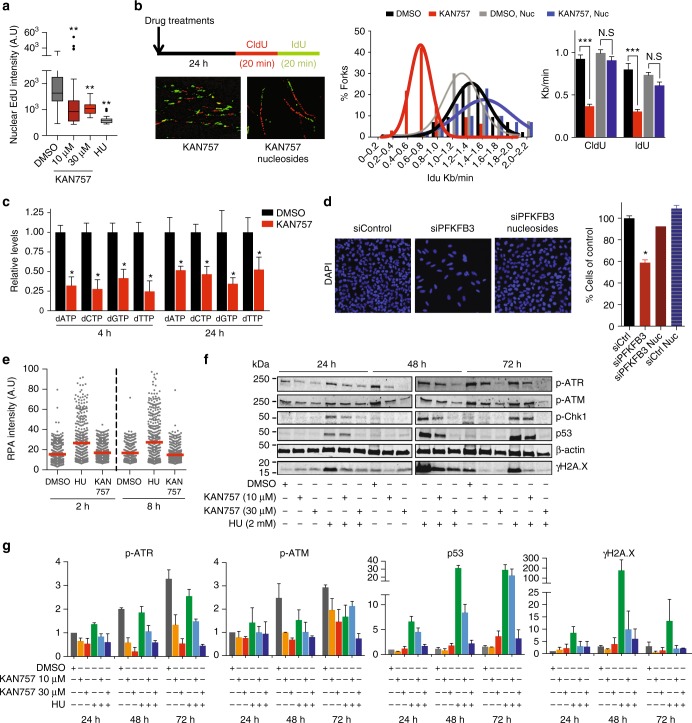
Nucleoside supplementation restores proliferation of PFKFB3 inhibited cells. **a** Effects on DNA synthesis of U2OS cells treated with DMSO or indicated concentrations of KAN757 (4 h), pulsed with 10 μM EdU for 40 min, followed by fixation, fluorescent labeling, and quantification in CellProfiler, >100 cells were analyzed per condition, *n* = 2. **P < 0.01; one-way ANOVA analysis. **b** Schematic representation of the DNA fiber assay and representative images of DNA strands incorporating CldU (red) and IdU (green) from U2OS cells that have received treatment with 10 μM KAN757 supplemented with or without 30 μM nucleosides. Graphs show the distribution of average fork speed and calculated mean fork speed for the indicated treatments (*n* > 100 forks per condition). Data are displayed as means ± SEM, *n* = 3. ***P < 0.001; Student’s *t*-test. **c** dNTP measurements of U2OS cells treated as indicated. Relative levels of dNTPs were calculated relative to DMSO. Data are displayed as mean ± SEM, *n* = 2. *P < 0.05; Student’s *t*-test. **d** Metabolic rescue of the proliferation of U2OS cells following 10 nM siControl or siPFKFB3 treatments and supplementation with or without 30 μM nucleosides was assessed by DAPI staining and counting nuclei using CellProfiler. siControl cells are set as reference cells. Data are displayed as means ± SEM, *n* = 2. *P < 0.05; one-way ANOVA analysis. **e** Scatter dot plot representing the nuclear intensity of the fluorescence levels of RPA32 in U2OS cells following treatment with DMSO or indicated inhibitors as quantified using CellProfiler. Lines represent mean fluorescence, *n* > 500 cells/treatment. **f** Representative immunoblots (of *n* = 2) of U2OS cells treated with DMSO, KAN757 (10, 30 µM) and/or HU (2 mM) for indicated time points. Bands correspond to whole cell extracts, β-actin was used as loading control. **g** Bar charts representing relative levels of phosphorylated ATR and ATM, p53 and γH2AX in (**f**). Densitometric analysis was performed using Image Studio^TM^ Lite Software. Signal intensity data were normalized against β-actin and then normalized to DMSO 24 h. Data are displayed as means ± SD, *n* = 2 independent experiments. KAN757 = KAN0438757

Depletion of dNTP pools through inhibition of the RNR is well known to stall replication forks that eventually collapse and generates DSBs^41^. Thus we sought to determine whether inhibition of PFKFB3 also in the absence of IR would modulate deoxynucleotide incorporation in RNR-dependent processes. Indeed, we observed decreased EdU intensity per cell in the EdU-positive cell population upon KAN0438757 treatment (Fig. 6a), indicative of impaired DNA replication. Notably, the decrease in EdU intensity upon PFKFB3 inhibition was similar in magnitude to that observed for inhibition of replication and induction of replication stress by hydroxyurea (HU) (Fig. 6a). To further investigate whether DNA replication is limited by insufficient salvage of deoxynucleotides in cells treated with PFKFB3 inhibitor, we performed DNA labeling studies to directly determine nucleotide incorporation into DNA over time by using the DNA fiber technique. KAN0438757 treatment severely decreased incorporation of 5-chloro-2′-deoxyuridine (CldU) and 5-iodo-2′-deoxyuridine (IdU) in U2OS cells (Fig. 6b), thus the speed of the replication fork is impaired upon PFKFB3 inhibition. Notably, nucleoside supplementation during the duration of KAN0438757 treatment restored the replication fork speed (Fig. 6b). To further confirm a role for PFKFB3 in intracellular dNTP supply, we next measured the dNTP pools upon KAN0438757 treatment of U2OS cells at the time points for the decreased EdU intensity (4 h) as well as for the inhibition of replication fork speed (24 h). KAN0438757 treatment resulted in a 50–75% decrease for all four dNTPs (Fig. 6c). This together with the nucleoside rescue of fork speed suggests that reduced dNTP pools could explain the decreased fork progression upon PFKFB3 inhibition. To complement the PFKFB3 inhibitor data, we supplemented cells with nucleosides at the timepoint for siRNA transfections and observed restored proliferation of the PFKFB3 knockdown cells (Fig. 6d).

We next investigated if the stalled forks upon KAN0438757 treatment collapse as measured by quantification of ssDNA coated by RPA. In contrast to HU, PFKFB3 inhibition did not induce RPA-coated ssDNA, p53 induction, or phosphorylation of either ATR, ATM, Chk1, or H2AX (Fig. 6e–g). Combined inhibition of PFKFB3 and RNR blocked the HU-dependent induction of phosphorylated ATR, Chk1, and ATM, increase in p53 levels as well as an increase in γH2AX levels (Fig. 6f, g), in line with PFKFB3 activity being upstream of RRM2 recruitment (Fig. 5b). Our data suggests that PFKFB3 inhibition results in the insufficient supply of nucleotides during replication, resulting in stalled replication forks without causing replication fork collapse or checkpoint activation.

## Discussion

Targeting a metabolic enzyme that is only needed for functional DDR in cancer cells and not in normal cells could be used to selectively impair survival of cancer cells and avoid toxicity to non-cancer cells. In this study, we identify PFKFB3 as a novel component of the DNA damage response and repair, demonstrate PFKFB3 activity as essential for DNA replication as well as generate a selective PFKFB3 inhibitor KAN0438757.

**Fig. 7:**
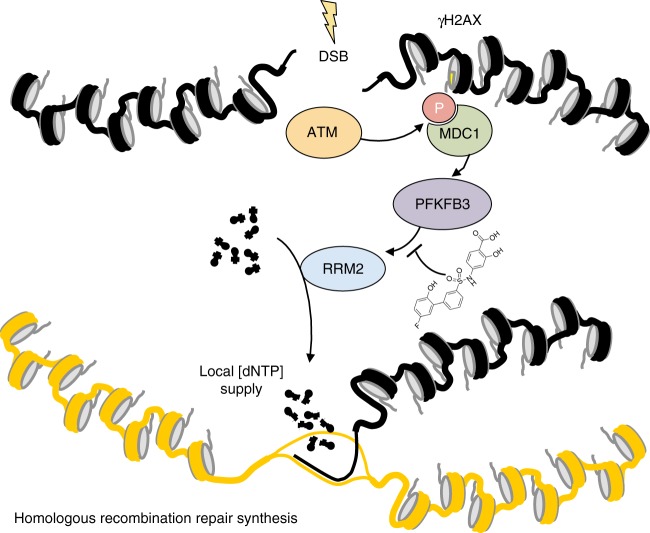
PFKFB3 mediates ATM-dependent break-induced dNTP supply. MRE**-**ATM-γH2AX-MDC1 signaling recruits PFKFB3 into nuclear foci, within the DNA damage response upon IR. PFKFB3 activity is required for subsequent recruitment of the RRM2 subunit of ribonucleotide reductase to sites of DNA damage to potentially generate a local dNTP pool. Inhibition of PFKFB3 activity impairs IR-induced repair synthesis, homologous recombination, and replication fork progression, which is restored by nucleoside supplementation

Our data strongly support a model where PFKFB3 is recruited rapidly upon IR through the MRN complex-ATM-γH2AX-MDC1 into nuclear foci at DNA damage and repair sites. Consequently, PFKFB3 is required for recruitment of the downstream HR repair factors BRCA1, RPA, and RAD51 as well as mobilization of the RRM2 subunit of RNR to sites of DNA damage (Fig. 7). Inhibition of PFKFB3, by siRNA silencing or KAN0438757, blocks HR activity and disrupts deoxynucleotide incorporation during DNA repair. This result in unrepaired DSBs, consistent with high levels of residual γH2AX foci, delayed recovery from IR-induced G2/M phase arrest and induces radiosensitization.

The intracellular levels of dNTPs are under tight control and increase 20-fold during the replicative S phase and upon DNA damage^42^. Consequently, imbalanced dNTP pools result in impaired replication fork speed and defective DNA repair^43,44^. Thus, a significant contribution to the impairment in DNA repair and replication upon PFKFB3 inhibition is likely coming from defective nucleotide incorporation; supported by dNTP measurements, IR-induced EdU incorporation studies in the G2/M phase of the cell cycle, as well as nucleoside rescue experiments. The control of the dNTP pools is exerted mainly via the RNR enzyme, with the RRM2 subunit being recruited to DNA damage sites^39,40,45^. Consistent with this, we identified the interaction between PFKFB3 and RRM2 upon IR as well as co-localization in a kinase-dependent manner upon induction of DSBs. The benefit of this recruitment could be to compartmentalize dNTPs directly at the DNA lesions to allow a high local dNTP concentration upon DNA repair. However, the limitation in determining dNTP concentrations site specifically in the nucleus makes it difficult to assess. Therefore we here assessed IR-induced EdU incorporation in the G2/M phase upon IR as well as total dNTP levels upon PFKFB3 inhibition both of which decreased upon PKFB3 inhibition. Our findings suggest that via recruitment of RRM2, PFKFB3 ensures local dNTP supply at DSB sites in a timely manner to coordinate the efficient supply of dNTPs with the recruitment of HR factors upon repair (Fig. 7).

Our data places PFKFB3 downstream in the IR-induced ATM signaling pathway (Fig. 7). Previously it has been reported that ATM-defective patients are insulin resistant and display symptoms of altered glucose metabolism^46^. Consistent with this, proteomic profiling of ATM proficient versus deficient lymphoblastoid cells revealed an altered metabolism as well as enrichment of proteins involved in glycolysis/gluconeogenesis pathway and carbohydrate metabolism^47^. The advantage of ATM pushing metabolic alterations could be to provide precursors such as nucleotides and amino acids for biosynthetic reactions to rapidly fuel cells upon DNA damage to promote repair, for instance providing the cell with ATP.

Although it is easy to anticipate that a possible PFKFB isoform switch during cancer transformation to PFKFB3 would give the advantage of a high glycolytic flux in malignant cells due to the enzyme’s high kinase to phosphatase ratio, nuclear PFKFB3 has been shown to drive proliferation dependent on its enzymatic function without increasing the overall cellular glycolysis^22^. Notably, no differential glucose consumption can be measured in vitro upon IR exposure^48^. Although the glycolytic enzyme phosphoglycerate mutase 1 (PGAM1) has been implicated in supporting HR and modulate the dNTP pool via its enzymatic activity, PGAM1 does not (and no glycolytic enzyme has to our knowledge been shown to) relocalize into repair foci in the nuclei upon DSB induction to support HR^49^. Thus the PFKFB3-mediated regulation of DNA repair is most likely not due to a global upregulation of glycolysis upon IR, suggesting a possible localized enzymatic function of PFKFB3 to support DNA repair and replication in the nucleus, likely by providing local dNTPs. However, a limitation of this study is that we were unable to measure synthesis of fructose-2,6-bisphosphate at DSB repair sites. To determine the extent to which local fructose-2,6-bisphosphate contributes to PFKFB3-mediated DNA repair, the focus of future research should be directed towards the development of sensitive tools capable of detecting fructose-2,6-bisphosphate directly at DNA repair sites.

The PFKFB3 isoform is favored in malignant cells with its enzymatic activity following its expression, thus its enzymatic activities represent an attractive anti-cancer drug target. Here, we report a radiosensitizing effect upon PFKBF3 reduction and inhibition, as demonstrated by suppression of HR and a high frequency of unrepaired DNA damage, as measured by assessing γH2AX levels. Consistent with its expression profile, the radiosensitizing effect upon PFKFB3 inhibition seems selective to cancer cells as survival of immortalized cells was only marginally affected at the doses affecting the survival of cancer cells upon IR. In the clinic, the radiation dose set to kill cancer cells in patients is limited with respect to normal tissue tolerance, thus our findings suggest PFKFB3 inhibition as an attractive approach to potentially achieve cancer-specific DNA repair deficiency upon radiation therapy. Several inhibitors targeting enzymes involved in DNA repair are under clinical evaluation^50^; with our data in mind demonstrating a tight association between PFKFB3 activity and successful DNA repair, it is easy to envision that combining such inhibitors with our PFKFB3 inhibitor could improve the therapeutic index further.

## Methods

### Cells and culture conditions

U2OS, PANC-1, NUGC-3, SW620, and MIA PaCa-2 were obtained from American Type Culture Collection (ATCC, Manassas, VA, USA). The BJ-hTER and BJ-RasV12 cell were provided by W. Hahn (Dana-Farber Cancer Institute) and the DR-GFP U2OS cells were provided by M. Jasin (Memorial Sloan Kettering Cancer Center). U2OS, DR-GFP U2OS, BJ-hTERT, and BJ-RasV12 cells were maintained in Dulbecco’s modified Eagle medium (DMEM) supplemented with 5% fetal bovine serum (FBS, Life Technologies/ThermoFisher Scientific), and penicillin–streptomycin antibiotics (50 U/mL). MIA PaCa-2, PANC1, SW620, and SW480 were grown in DMEM (Lonza Biosciences), with 10% FBS (Life Technologies/ThermoFisher Scientific). Cells were grown at 37 °C in 5% CO_2_ humidified incubators.

### Clonogenic survival

U2OS cells were transfected with indicated siRNAs and 24 h later exposed to IR (2 Gy) or left untreated and allowed to recover for 4 h. One-hundred U2OS cells were then seeded into 6-well plates and after 10 days colonies were fixed and stained with 4% methylene blue (Sigma Aldrich) in methanol. BJ TERT (500 cells), BJ RAS (200 cells), or U2OS (200 cells) were seeded into 6-well plates 24 h prior to treatment with inhibitors as indicated or vehicle for 24 h and then subjected to IR (2 Gy). The inhibitors were washed out 72 h later and colonies were fixed 4 days (BJ RAS) or 7 days (BJ TERT) later with 4% methylene blue in methanol.

For RAD51 rescue experiments, 1 × 10^6^ BJ RAS cells were seeded in 100 mm dishes and transfected the next day with 5 μg empty vector or RAD51-GFP plasmid. After 24 h, 200 cells were seeded on 6-well plates, at the time of seeding equal amounts of cells were collected from RAD51 and empty vector-transfected cells, and lysed and subjected to western blot to confirm the overexpression of RAD51. Twenty-four hours post-seeding, cells were treated with inhibitors as indicated or vehicle for 24 h and then exposed to IR (2 Gy). The inhibitors were washed out 72 h later and colonies were fixed 12 days later with 4% methylene blue in methanol. Colonies were counted manually.

### Ionizing radiation

γ-Irradiation was performed with a 137Cs source (Scanditronix) at the Karolinska Institutet, Stockholm, at a photon dose rate of 0.5 Gy/min. Dosimetry was done with an ionization chamber as well as with ferro-sulphate. X-ray high-intensity radiation was performed with an X-RAD 225 XL irradiator (Precision X-Ray), courtesy of the Farnebo lab (Cancer Center Karolinska).

### Immunofluorescence microscopy

Cells were grown on sterilized coverslips and fixed with 4% paraformaldehyde (PFA) (Santa Cruz) and 2% sucrose (Sigma Aldrich) for 15 min at room temperature. They were then permeabilized with 0.2% NP-40 (Thermo Scientific) for 10 min at room temperature followed by 30 min of blocking in blocking buffer (2 or 3% BSA, 5% glycerol, 0.1% Tween 20 (Sigma Aldrich)). Coverslips were subsequently incubated for 1, 2, or 24 h in primary antibody and 40 min in secondary antibody diluted in blocking buffer, DNA was stained with DAPI for 10 min. The coverslips were mounted with Prolonged Gold mounting medium (Invitrogen). Images were acquired with a Zeiss LSM 780 microscope using a 40× or 63× oil immersion lens and processed in ImageJ and CellProfiler.

### In situ cell fractionation

To visualize PFKFB3, RPA32, RAD51, BRCA1, 53BP1, and RRM2 IRIF, the cells were first washed with PBS and then incubated for 5 min at room temperature with cytoskeleton (CSK) buffer containing 10 mM PIPES pH 7.0 (Sigma Aldrich), 100 mM NaCl (EMD Millipore), 300 mM sucrose (Sigma Aldrich), 3 mM MgCl_2_ (Sigma Aldrich), and 0.7% Triton X-100 (Sigma Aldrich). After these treatments, the cells were washed once again with PBS and then fixed in 4% PFA with 2% sucrose.

### Western blot

Cells were lysed on ice using the NP-40 lysis buffer (50 mM Tris–HCl pH 8.0, 150 mM NaCl, 1% NP-40) supplemented with protease and phosphatase inhibitors (Roche). Protein lysates were quantified using the Pierce BCA Protein Assay Kit (ThermoFisher Scientific), equal amounts of protein were heated to 70 °C in NuPAGE LDS Sample Buffer (ThermoFisher Scientific) and loaded on Mini-PROTEAN® precast gels (Bio-Rad) and run at 120 V. Transfer to nitrocellulose membrane (Bio-Rad) was done using Trans-Blot Turbo Transfer System (Bio-Rad). Membranes were blocked for 1 h at RT in blocking buffer (5% BSA, 0.1% Tween-20 in PBS, or 1:1 Li-Cor Blocking Buffer/TBS + 0.05% Tween-20 (TBS-T)), incubated with primary antibodies overnight at 4 °C and then incubated 1 h at RT with secondary antibodies diluted at 1:10,000 in 1:1 Li-Cor blocking buffer/TBS-T. Bands were visualized with an Odyssey Fc Imager and analyzed with ImageJ or with Image Studio Software (Li-Cor Biosciences). Uncropped western blots are shown in Supplementary Figure 10, 11, 12, 13, 14, 15, 16.

### Immunoprecipitations

U2OS cells were seeded into 6-well plates 24 h before transfection with 1 μg of indicated plasmids, after 24 h post-transfection cells were exposed to IR (6 Gy) or left untreated. Two hours post-IR, cells were assayed for protein interactions as previously described^51^. The supernatant was immunoprecipitated with RRM2 antibody (Santa Cruz, catalog no. sc10844) or IgG overnight at 4 °C. The following day, 40 μl Protein G slurry was added for 4 h at 4  °C, then protein complexes were washed four times in 500 μl lysis buffer, and lysed in 60 μl NuPAGE LDS Sample Buffer (ThermoFisher Scientific). Protein complexes were subjected to 10% SDS-PAGE followed by western blot analysis with FLAG antibody (Sigma Aldrich, catalog no. F3165).

### Antibodies

The following antibodies were used in immunofluorescence and Western blots: rabbit anti-PFKFB3 (Proteintech, cat. no. 13763-1-AP, WB 1:500, IF 1:100), rabbit anti-PFKFB3 (Cell Signaling, cat. no. 13123, 1:500), rabbit anti-ATM p-Ser-1981 (Abcam, cat. no. ab81292, 1:500), mouse anti-ATM (Santa Cruz, cat. no. sc-23921, 1:500), rabbit anti-ATR p-Ser-428 (Cell Signaling, cat. no. 2853S, 1:500), rabbit anti-DNA-PK p-Ser-2056 (Abcam, cat. no. ab18192, 1:500), mouse anti-Flag (Sigma Aldrich, cat. no. F3165, 1:500), rabbit anti-KAP1 p-Ser-824 (Bethyl Laboratories, cat. no. A300-767A, 1:500), goat anti-KAP1 (Bethyl Laboratories, cat. no. A303-838A, 1:500), mouse anti-γ-H2AX (Millipore, cat. no. 05-636, WB 1:1000, IF 1:500), rabbit anti-γ-H2AX (Cell Signaling, cat. no. 2577 S, 1:1000), rabbit anti-Histone H2A (Abcam, cat. no. ab18255, 1:500), rabbit anti-Histone H3 (Abcam, cat. no. ab1791, 1:2000), rabbit anti-Mre11 (Novus Biologicals, cat. no. NB100-142, 1:500), rabbit anti-Nbs1 (Novus Biologicals, cat. no. NB100-143, 1:500), rabbit anti-RAD51 (Santa Cruz, cat. no. sc-8349, 1:500), mouse anti-RAD51 (Abcam, cat. no. ab213, 1:500), rabbit anti-RPA p-Ser-33 (Novus Biologicals, cat. no. NB100-544, 1:500), rat anti-RPA2/32 (Cell Signaling, cat. no. 2208S, 1:100), rabbit anti-53BP1 (Novus Biologicals, cat. no. NB100-904, 1:500), mouse anti-53BP1 (Abcam, cat. no. ab36823, 1:100), mouse anti-BRCA1 (Santa Cruz, cat. no. sc-6954, 1:100), goat anti-RRM2 (Santa Cruz, cat. no. sc-10844, 1:500), mouse anti-RRM2 (Abcam, cat. no. ab57653, 1:500), mouse anti-RRM2 (Sigma Aldrich, cat. no. WH0006241M1, WB 1:500, IF 1:100), mouse anti-p53 (Santa Cruz, cat. no. sc-126, 1:500), rabbit anti-Chk1 p-Ser-345 (Cell Signaling, cat. no. 2341S, 1:250), rabbit anti-MDC1 (Abcam, cat. no. ab11169, 1:500), mouse anti-SOD-1 (Santa Cruz, cat. no. sc-17767, 1:1000), rabbit anti-GFP (Santa Cruz, cat. no. sc-8334, 1:1000), mouse anti-β-actin (Abcam, cat. no. ab6276, 1:10,000), rabbit anti-α-tubulin (Abcam, cat. no. ab18251, 1:1000), rabbit anti-β-tubulin (Abcam, cat. no. ab6046, 1:1000), and goat anti-vinculin (Santa Cruz, cat. no. sc-7649, 1:1000).

The secondary antibodies for confocal were: goat anti-rabbit Alexa Fluor 488 (Invitrogen, cat. no. A11008, 1:500), goat anti-mouse Alexa Fluor 488 (Invitrogen, cat. no. A11029, 1:500), donkey anti-mouse Alexa Fluor 488 (Invitrogen, cat. no. A-21202, 1:500), goat anti-rat Alexa Fluor 555 (Invitrogen, cat. no. A-21434, 1:500), donkey anti-goat Alexa Fluor 555 (Invitrogen, cat. no. A-21432, 1:500), goat anti-rat Alexa Flour 568 (Invitrogen, cat. no. A-11077, 1:500), donkey anti-goat Alexa Fluor 633 (Invitrogen, cat. no. A-21082, 1:500), donkey anti-mouse Alexa Fluor 647 (Invitrogen, cat. no. A-3157, 1:500).

The secondary antibodies for Western blot were: donkey anti-mouse IgG IRDye 680RD (925-68072, 1:5000), goat anti-rabbit IgG IRDye 800CW (925-32211, 1:5000), donkey anti-rabbit IRDye 800CW (926-32213, 1:5000), donkey anti-mouse IRDye 800CW (926-32212, 1:5000), and donkey anti-goat IRDye 800CW (925-32214, 1:5000) from Li-Cor Biosciences.

### EdU labeling of cultured cells

U2OS cells were seeded on sterilized slides and treated with 10 or 30 μM of 757 for 6 h, or 2 mM of hydroxyurea (HU) for 3 h (DMSO was used as control vehicle). Last 40 min of treatment, 10 μM of the thymidine analogue 5-ethynyl-2′-deoxyuridine (EdU) (Sigma Aldrich, catalog no. 1T511285) was added; cells were washed with PBS and then fixed with paraformaldehyde 4% and sucrose 2%. For the detection of incorporated EdU, the Click IT reaction was performed in accordance with the supplier’s recommendations (Life Technologies/ThermoFisher Scientific). Images were acquired with a Zeiss LSM 780 microscope using a 40× oil immersion lens and processed in ImageJ and CellProfiler (version 2.2.0).

### siRNA transfections

The siRNA oligonucleotides used were siPFKFB3#1 (Ambion, 4390824, ID:s10357), siPFKFB3#2 (Ambion, 4390824, ID:s10358), siH2AX (Qiagen, SI00032844), siMDC1 (Dharmacon, L-003506-00-0005), siRRM2 (Dharmacon, L-010379-00-0005), siRAD51 (Qiagen, SI02663682), and siControl (Dharmacon). siRNA (10 nM) was transfected into cells using INTERFERin (Polyplus transfections) transfection reagent in accordance with the supplier’s recommendations.

### Plasmid transfections

The I-SceI plasmid was kindly provided by M. Jasin (Memorial Sloan Kettering Cancer Center), RAD51-GFP was a kind gift from F. Dantzer (Institut de Recherche de l’Ecole de Biotechnologie de Strasbourg), and empty vector and FLAG-PFKFB3 has previously been described^22^. Plasmids were transfected into cells using JetPEI or JetPRIME (Polyplus transfections) transfection reagents in accordance with the supplier’s recommendations.

### Treatment with small-molecule inhibitors and metabolites

3-PO, ATM (KU55933^52^), and DNA-PK (NU7441^53^) inhibitors were obtained from TOCRIS bioscience. The ATR inhibitor (VE-821^54^) was obtained from Axon MedChem (catalog no. Axon 1893) and Hydroxyurea (HU) and the MRN complex inhibitor Mirin (catalog no. M9948) were purchased from Sigma Aldrich. Where appropriate, 10 μM ATM inhibitor, 2 μM DNA-PK inhibitor, 2.5 μM ATR inhibitor, and 2 mM HU were added to the culture medium 6 h prior to IR. For experiments using Mirin, 300 μM was added to the culture medium 2 h prior to IR. A 30 mM nucleoside mix containing adenosine, cytidine, guanosine, and uridine (Sigma Aldrich) was prepared fresh before each experiment and added to a final concentration of 30 μM at the same time as siRNA transfections or inhibitor treatments.

### Flow cytometry

For cell cycle analysis, cells were treated or transfected with siRNA as described and harvested at the indicated time points with trypsin. For studies using IR, cells were exposed to IR with 2 Gy, and harvested at the indicated time points with trypsin. The cells were washed in PBS and fixed with 70% ethanol in PBS at 4 °C for 1 h and then stored at −20 °C until staining. Ethanol was removed by centrifugation and cells were then washed in PBS before staining DNA with propidium iodide (PI) solution containing 40 μg/mL PI, 100 μg/mL RNase A, and 0.1% Triton X-100 (Sigma Aldrich). Cells were analyzed by flow cytometry on a FACS Navios (Beckman Coulter) using Kaluza software.

### Method for measurement of intracellular dNTP

HIV-1 RT-based dNTP assay^55^ was used to quantify dNTP levels in U2OS cells upon indicated treatment and time points. Briefly, cells were trypsinized and counted before dNTPs extraction by 60% methanol solution, the dried dNTP samples were resuspended with water, and then RT-based primer extension reaction was performed to determine the amounts of dNTPs. Obtained dNTP amounts were normalized to 1 × 10^6^ cells, and dNTP levels were related to control treatment with DMSO.

### DR-GFP (HR) assays

5 × 10^4^ U2OS (DR-GFP) cells were seeded into 12-well plates, 24 h later cells were treated with the indicated siRNAs or inhibitors and, 24 h later, transfected with 150 ng of an I-SceI vector using JetPEI or JetPRIME (Polyplus transfections). The next day, the medium was changed; 24 h after this, cells were harvested by trypsinization and washed with PBS, and the GFP signal was measured by flow cytometry on a FACS Navios (as described above). The frequency of repair in cells treated with inhibitors or transfected with the various siRNAs was calculated relative to cells treated with DMSO or transfected with control siRNA.

### DNA fiber assay

1 × 10^5^ cells were seeded into 6-well plates, 24 h later cells were treated with the indicated inhibitors or nucleosides, and 24 h later DNA was labeled with 25 μM CldU (Sigma, C6891) in pre-warmed DMEM for 20 min, washed and then labeled with 250 μM IdU (Sigma I7125) in pre-warmed DMEM for 20 min, before harvesting by scraping in cold PBS. Harvested cells were diluted to 1 × 10^6^ cells/mL, spreading and staining was performed as described previously described^56^. Fluorescence images were captured using a Zeiss LSM 780 inverted confocal microscope using a 63 × 1.4 oil immersion objective, excitation wavelengths of 488 and 555 nm, and analyzed using the ImageJ software. At least 300 unidirectional forks labeled with both CldU and IdU were measured for every condition.

### CETSA

U2OS cells in exponential growth rate were treated with indicated inhibitors at a concentration of 10 μM or DMSO for 6, 24, or 72 h and then harvested using trypsin, upon detachment trypsinization was inhibited by addition of cell media and cells spun down at 200 × *g* for 10 min at 4 °C. The pellet was resuspended in TBS (50 mM Tris–HCl (pH 7.5) and 150 mM NaCl) supplemented with protease inhibitors (Roche), cells were lysed by a freeze–thaw circle three times at −80 °C for 3 min and then 37 °C for 3 min. Debris was removed by centrifugation at 16,000 × *g* for 20 min at 4 °C, cell lysates were aliquoted and heated to indicated temperatures for 3 min. Insoluble proteins were then removed by centrifugation at 16,000 × *g* at 4 °C for 20 min, supernatant was transferred to new tubes and protein concentration was determined using BCA assay (Pierce). Western blot was performed according to standard procedures.

### Gene expression profiling

Expression profiling of PFKFB3 mRNA levels (GEO accession number GSE13280) in mononuclear cells isolated from bone marrow samples from pediatric B-precursor ALL patients responsive to radiotherapy before (*n* = 11) and after (*n* = 11) radiotherapy, pediatric ALL patients resistant to radiotherapy before (*n* = 11) and after (*n* = 11) radiotherapy. Tukey’s multiple comparisons test was used to calculate significance (GraphPad Prism).

### Statistical analysis

Statistical significance was determined via two-tailed Student’s *t*-test or ANOVA using GraphPad Prism 7 or Excel. The results originate from at least two independent experiments and are presented as means ± standard error of the mean or standard deviation. Significance values were set at *P ≤ 0.05, **P ≤ 0.01, ***P ≤ 0.001.

### Chemical synthesis

NMR spectra were recorded on a Varian Inova 600 equipped with a triple resonance cold probe or a triple resonance probe. Purity was determined by analytical HPLC (Agilent Series 1100 system) using two different columns (ACE C8 (3 µm, 3.0 × 50 mm) column with 0.1% TFA in MilliQ water/MeCN as mobile phase and XTerra (3.5 µm, 3.0 × 50 mm) column with 10 mM pH10 NH_4_HCO_3_/MeCN as mobile phase). Electrospray mass spectrometry (ES-MS), used to support correct molecular weight identity for synthesized compounds, was performed using an Agilent 1100 Series Liquid Chromatograph/Mass Selective Detector (MSD) to obtain the pseudo molecular [M + H]^+^ ion of the target molecules. The compounds were named using the software ACD Labs.

Synthesis of 4-{[(5′-Fluoro-2′-hydroxybiphenyl-3-yl)sulfonyl]amino}-2-hydroxybenzoic acid (KAN0438241): A mixture of methyl 4-{[(3-bromophenyl)sulfonyl]amino}-2-hydroxybenzoate (1.05 g, 2.7 mmol), 5-fluoro-2-hydroxyphenylboronic acid (0.47 g, 3.0 mmol), DIPEA (1.05 g, 8.1 mmol), and Pd(dppf)Cl_2_.CH_2_Cl_2_ (44 mg, 54 µmol) in aqueous dioxane (30 mL dioxane, 5 mL water) was heated at 80 °C under N_2_ atmosphere overnight. Water and EtOAc were added. The organic phase was washed with 1 M HCl and brine, dried over MgSO_4_, filtered and concentrated. To the residue, 1 M NaOH (20 mL) was added. The reaction was stirred at room temperature for 3 h. A spoonful of charcoal was added. The mixture was stirred for 1 h and filtered through a pad of celite. The mother liquid was washed twice with CH_2_Cl_2_ and acidified with concentrated H_3_PO_4_. EtOAc was added. The organic phase was washed with 1 M HCl and brine, dried over MgSO_4_, filtered, and concentrated. The residue was recrystallized from water/MeOH. The title compound was obtained as a white solid (0.77 g, 70% yield, 100% purity). ^1^H NMR (600 MHz, Methanol-d_4_): δ ppm 8.11 (t, *J* = 1.7 Hz, 1H), 7.78–7.82 (m, 2H), 7.70 (d, *J* = 8.5 Hz, 1H), 7.56 (t, *J* = 7.8 Hz, 1H), 6.92–6.99 (m, 2H), 6.86–6.90 (m, 1H), 6.72 (d, *J* = 1.8 Hz, 1H), 6.68 (dd, *J* = 8.8, 2.1 Hz, 1H). MS (ESI+) *m*/*z* 404 [M + H]^+^.

Synthesis of 2-Hydroxyethyl-4-{[(5′-fluoro-2′-hydroxybiphenyl-3-yl)sulfonyl]amino}-2-hydroxybenzoate (KAN0438757): A mixture of 4-{[(5′-fluoro-2′-hydroxybiphenyl-3-yl)sulfonyl]amino}-2-hydroxybenzoic acid (0.020 g, 0.050 mmol), ethylene glycol (400 µL), and conc. H_2_SO_4_ was stirred at 80 °C for 1 day. The reaction mixture was diluted with MeCN and purified by preparative HPLC (Gilson HPLC system; ACE C8 (5 µm, 21 × 50 mm) column; 0.1%TFA in MilliQ H_2_O/MeCN as mobile phase; fractions were collected based on the UV-signal). The title compound was obtained in 100% yield (22 mg, 97% purity). ^1^H NMR (600 MHz, DMSO-d_6_): δ ppm 10.91 (s, 1H), 10.60 (s, 1H), 9.82 (s, 1H), 8.11 (t, *J* = 1.7 Hz, 1H), 7.83 (d, *J* = 7.6 Hz, 1H), 7.78 (d, *J* = 8.8 Hz, 1H), 7.71 (d, *J* = 8.5 Hz, 1H), 7.63 (t, *J* = 7.9 Hz, 1H), 7.12 (dd, *J* = 9.5, 3.1 Hz, 1H), 7.03–7.09 (m, 1H), 6.96 (dd, *J* = 9.2, 4.9 Hz, 1H), 6.73 (dd, *J* = 8.7, 2.0 Hz, 1H), 6.70 (d, *J* = 2.1 Hz, 1H), 4.90 (t, *J* = 5.6 Hz, 1H), 4.23–4.29 (m, 2H), 3.60–3.70 (m, 2H). ^13^C NMR (151 MHz, DMSO-d_6_) δ ppm 168.5 (s), 161.2 (s), 155.7 (d, *J* = 234.9 Hz), 150.7 (d, *J* = 1.5 Hz), 144.4 (s), 139.0 (s), 138.4 (s), 133.7 (s), 131.6 (s), 129.4 (s), 127.2 (s), 126.4 (d, *J* = 7.5 Hz), 125.2 (s), 117.2 (d, *J* = 8.0 Hz), 116.1 (d, *J* = 23.3 Hz), 115.8 (d, *J* = 22.4 Hz), 109.5 (s), 108.0 (s), 105.3 (s), 66.7 (s), 58.9 (s). MS (ESI+) *m*/*z* 448 [M + H]^+^.

KAN0438757 was also prepared on a 6-g scale according to a similar protocol with some minor changes, such as a lower temperature (50 °C for 1 week) and extractive workup (EtOAc).

### Isothermal titration calorimetry

ITC was performed for the titration of 200 μM KAN0438241 into 20 μM PFKFB3 protein. Raw ITC data was recorded for 12 × 3 μl injections (preceded by 0.4 μl injection). Data was collected with PEAQ-ITC system (MicroCal™, Malvern Instruments) at 25 °C, 10 μcal/s Reference Power, High Feedback, and 750 rpm stirring rate. A constant value of the heat of dilution was fitted along with the binding parameters. Data analysis performed with dedicated PEAQ-ITC analysis software (MicroCal, Malvern Instruments).

### Methods for measurement of enzymatic activity

The kinase activity of the bi-functional enzyme was quantified based on the production of ADP and F-2,6-P_2_ from ATP by ADP-GloTM Kinase Assay (Promega). The assay was performed in two steps; first, after the kinase reaction, an equal volume of ADP-GloTM Reagent was added to terminate the kinase reaction and deplete the remaining ATP. Second, the Kinase Detection Reagent was added to simultaneously convert ADP to ATP and allow the newly synthesized ATP to be measured using a luciferase/luciferin reaction. The light generated was measured using a luminescence counter (1450 MicroBeta TriLux). The assay was performed in white 384-well plates (OptiPlate, 6007299, PerkinElmer) by consecutive additions of a test compound solution (0.1 µL, serial diluted in DMSO from compound DMSO stock solution and dispensed by acoustic dispensing from UV-star 384-well plate 7360153, VWR), enzyme solution (5 µL) and substrate (fructose-6-phosphate (F6P) and adenosine-5′-triphosphate (ATP)) containing solution (5 µL). Controls in the absence of inhibitor (uninhibited activity, only DMSO), 100 nM of an in-house inhibitor (QC, 50% inhibited kinase activity), and 49.5 µM of an in-house inhibitor (completely inhibited kinase activity) were included in the plate. Four reference dose–response curves, one of them being the QC compound, were included and serial diluted as the test compounds. The final concentrations of all reagents per well were 50 mM Tris–HCl at pH 8.0, 10 mM MgCl_2_, 5 mM NaP_i_ at pH 8.0, 81.48 nM PFKFB3 or 145 nM PFKFB4, 8 µM ATP, 100 µM F6P (acid treated and then neutralized to remove any contaminating F-2,6-P2^34^), 0.005% Tween-20 and 1 mM dithiothreitol. Enzyme, compounds, and controls were pre-incubated at room temperature for 15 min before the addition of substrate solution. The enzymatic reaction was allowed to proceed for 20 min and the reaction was terminated by the addition of ADP-GloTM Reagent (10 µL) to all wells followed by 40 min incubation at room temperature. After addition of Kinase Detection Reagent (20 µL) to all wells, the plate was incubated for 30 min at room temperature, followed by measurement in 1450 MicroBeta TriLux. All plates were centrifuged after each addition. As described above, controls were included on each plate to define the values for uninhibited and fully inhibited reactions and these values were used to calculate the % inhibition of the enzymatic reaction at any given compound concentration. The inhibitory potency or IC_50_ values of test compounds on the kinase activity of the enzymes were calculated using a four-parameter model (model 205) in XLfit (IDBS ActivityBase XE Runner).

### Method for quantification intracellular F-2,6-P_2_

Quantification of F-2,6-P_2_ was performed as described by Van Schaftingen et al.^34^. The assay is based on the potent activation of pyrophosphate-dependent phosphofructokinase-1 (PPi-PFK) from potato tubers by F-2,6-P_2_. The use of a series of coupled enzymes leads to a consumption of nicotinamide adenine dinucleotide (NADH) that can be followed spectrophotometrically. PANC-1, NUGC-3, SW620, or MIA PaCa-2 cells were seeded at a density of 2.5 × 10^4^–3.5 × 10^4^ cells per well (96-well, CLS3595, Sigma-Aldrich) and incubated overnight at 37 °C and 5% CO_2_. Following day growth medium was replaced with starvation medium, DMEM/F12 without phenol red and glucose-free (SVA) supplemented with 0.25% FBS (Invitrogen). The plates were incubated for 18 h at 37 °C and 5% CO_2_. After 18 h of starvation, the cells were induced with compound or control solutions (100 µL). Compounds were either tested in two concentrations (50 and 10 µM) or in dose–response curves (starting from 50 µM), a dose–response curve of a reference inhibitor was included and all compounds were tested on duplicate plates. Compounds (in 96-well plates CLS 3365, Sigma-Aldrich) were serial diluted in DMSO with Janus automated liquid handling workstation (PerkinElmer). Compounds were transferred to a Greiner deep well plate (736-0155, VWR) with starvation medium. The start concentration of compounds in the dilution plate was 100 µM, 1% DMSO. For compounds tested as single points, 10 mM compound solutions were diluted 5-fold in DMSO to 2 mM, followed by the transfer to separate dilution plates with starvation medium. The final concentrations of compounds in these dilution plates were 100 or 20 µM, respectively, and final concentration of DMSO was 1%. The plates were incubated at 37 °C and 5% CO_2_ for 1 h, followed by the addition of 20 mM D-glucose (10 µL) in starvation medium. Controls, with and without glucose (1 mM), were included. After 2 h of incubation, the supernatants were discarded and the cells lysed by the addition of 250 mM NaOH (25 µL). The plates were incubated at 37 °C and 5% CO_2_ for 5 min followed by an addition of MilliQ dH_2_O (75 µL). The supernatants were further diluted with 210 µL MilliQ dH_2_O to a final concentration of 20 mM NaOH. 200 µL were transferred to NUNC 96-well plates (VWR) and the plates were sealed and stored at −20 °C until analysis. Plates stored at −20 °C were thawed and the F-2,6-P_2_ quantification was initiated by transferring 40 µL from each well of the NUNC plate to the corresponding position in a transparent 96-well SpectraPlate-MB (PerkinElmer). In order to ensure that all F-2,6-P_2_ measured values were within the linear range of response (between 0 and 1 nM final concentration of F-2,6-P_2_^34^), the same sample volume of an in-house produced F-2,6-P_2_ standard was included on each plate. The following reagents were added per well: 50 mM Tris–acetate pH 8.0, 0.15 mM NADH, 2 mM Mg(OAc)_2_, 1 mM F6P (acid treated and then neutralized to remove any contaminating F-2,6-P^34^), 0.5 mM pyrophosphate, 0.45 U/mL aldolase, 5 U/mL triose phosphate isomerase, 1.7 U/mL glycerol-3-phosphate dehydrogenase, 0.01 U/mL pyrophosphate-dependent phosphofructokinase from potato tubers and 0.2 mg/mL bovine serum albumin. The coupled enzymatic reaction was allowed to proceed for 45 min at room temperature and the absorbance at 340 nm was continuously measured every 30 s (SpectraMax plate reader, Molecular Devices). The measured absorbance was proportional to the concentration of NADH, which in turn was proportional to the levels of F-2,6-P_2_ within the linear range. This was defined by the F-2,6-P_2_ controls. The IC_50_ values for test compounds were calculated using a four-parameter model (model 205) in XLfit (Excel) and in XLfit (IDBS ActivityBase).

### Measurement of inhibition of cell proliferation

Cell proliferation was assayed by quantifying total cellular protein levels after indicated treatments and incubation times using the Sulphorhodamine B kit, TOX6 (Sigma-Aldrich). MIA PaCa-2, PANC1, SW620, and SW480 cells were plated at a density of 6 × 10^3^ cells/well in 96-well plates (CLS3596, Sigma-Aldrich) and allowed to attach overnight. The following day, compounds were diluted in dose–response curve mode, required volume was transferred to assay medium to a maximal DMSO concentration of 0.1%. The diluted compounds were added to the cells with a starting dose–response concentration of 100 µM, all compounds were tested in duplicate plates. After 72 h of incubation with compounds, total protein per well was precipitated using TCA according to the manufacturer's instructions; the sulphorhodamine dye was added to the air-dried wells. After 20 min incubation at room temperature, the dye was discarded and the samples were gently rinsed with 1% HOAc until clear. After air drying, bound dye was solubilized in 10 mM Tris base, and the absorbance of dye was measured at 565 nm. To quantify growth, samples were collected also at *t* = 0 h, and the resulting absorbance was set to 100%. The measured effect on cancer cell proliferation was quantified as the ratio between 72 h value and the 0 h value in percent, and this value was subsequently divided with the 72 h 0.1% DMSO control sample in percent demonstrating the growth-inhibitory effect.

### Crystallization

Diffraction data have been collected on BL14.1 operated by the Helmholtz-Zentrum Berlin (HZB) at the BESSY II electron storage ring (Berlin-Adlershof, Germany)^57^. Indexing, reduction, and scaling of diffraction data were performed using XDS^58^. Crystallographic refinement and model building was performed using Refmac5 and Coot^59–61^. Structure quality was assessed using MolProbity^62^. We refer to Supplementary Table 1 for crystallographic data collection and refinement statistics.

## Electronic supplementary material

Supplementary Information

Description of Additional Supplementary Files

Supplementary Dataset 1

## Data Availability

All of the structural data from this study have been submitted to the pdb database (PDB ID 6ETJ). All other supporting data from this study are available from the article and Supplementary Information files, or from the corresponding author upon reasonable request.
