# Supplementary material for: Targeting PFKFB3 radiosensitizes cancer cells and suppresses homologous recombination
Source: Nat Commun. 2018 Sep 24;9:3872. doi: 10.1038/s41467-018-06287-x (PMC6155239; doi:10.1038/s41467-018-06287-x)
Supplement: Supplementary file 1 — Supplementary Information [file 41467_2018_6287_MOESM1_ESM.pdf]

SUPPLEMENTARY INFORMATION

Targeting PFKFB3 radiosensitizes cancer cells and suppresses homologous recombination

Nina M.S. Gustafsson et al.

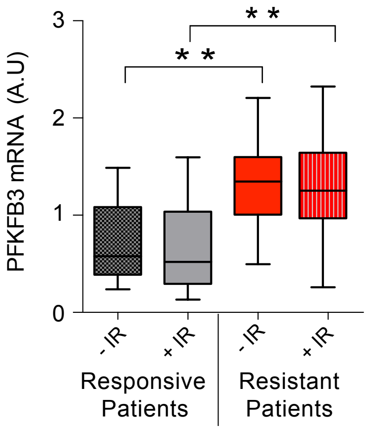

Supplementary Figure 1. Expression profiling of PFKFB3 mRNA levels in radiotherapy resistant and responsive patients.

Expression profiling of PFKFB3 mRNA levels (GEO accession number GSE13280) in mononuclear cells isolated from bone marrow samples from pediatric B-precursor ALL patients responsive to radiotherapy before (n=11) and after (n=11) radiotherapy, pediatric ALL patients resistant to radiotherapy before (n=11) and after (n=11) radiotherapy. Tukey's multiple comparisons test was used to calculate significance, ** $P<0.01$.

Supplementary Table 1. Crystallographic data collection and refinement statistics

**Data collection**	**PFKFB3**
Space group	$P6_522$
Cell dimensions	
a, b, c (Å)	103.77, 103.77, 259.56
α, β, γ (°)	90, 90, 120
Resolution (Å)	34.28-2.51 (2.58-2.51)
$I/\sigma I$	11.6 (1.9)
R_{meas}	11.1 (86.3)
Completeness (%)	99.5 (99.9)
Redundancy	4.1 (4.3)
Radiation source	BESSY II, BL14.1
Wavelength (Å)	0.9184
**Refinement**	
Resolution (Å)	34.28-2.51 (2.58-2.51)
No. reflections	27596
R/R_{free}	0.192/0.235
No. atoms	
Protein	3559
Ligands	75
Solvent	102
B-factors (Å^2)	
Protein	42.2
Ligands	45.6
Water	37.3
R.m.s. deviations	
Bond lengts (Å)	0.011
Bond angles (°)	1.626
Ramachandran plot (%)	
Favored region	96.7
Allowed region	3.3
Outlier region	0.0

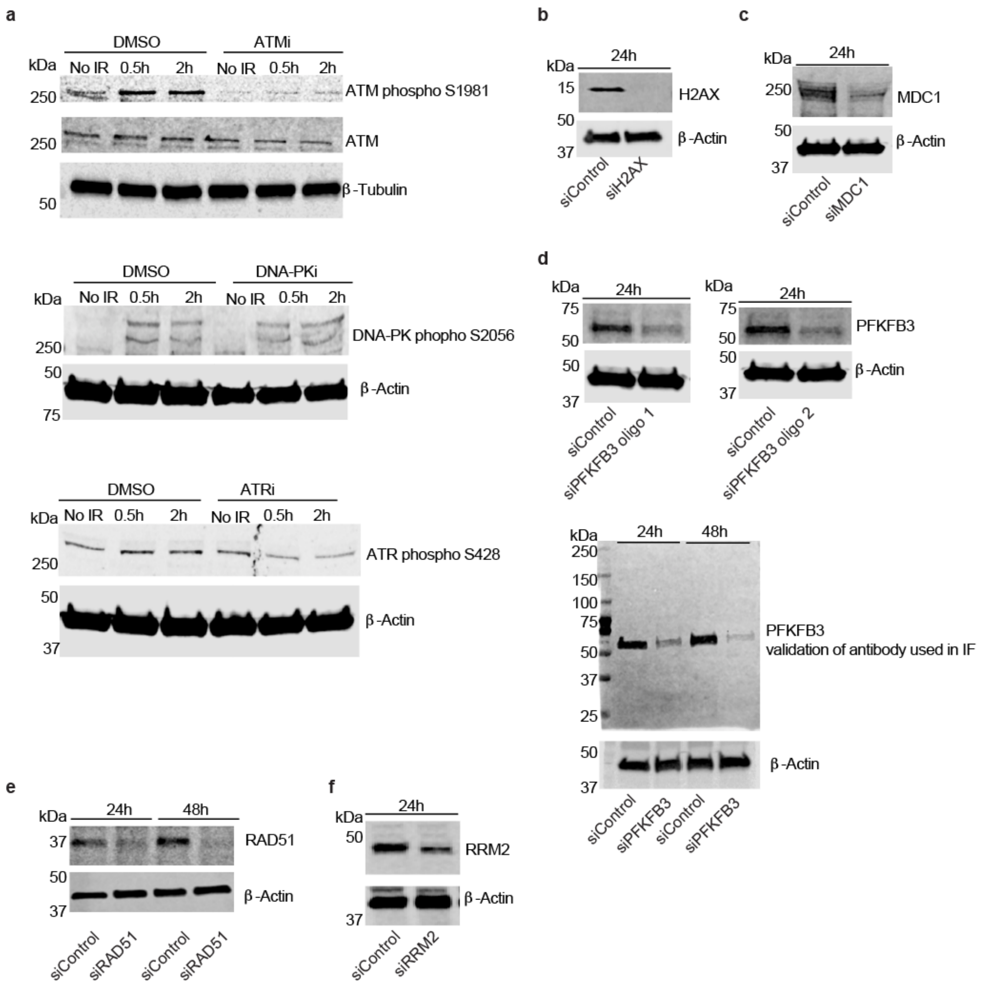

Supplementary Figure 2. Confirmation of siRNA knockdowns.
Representative immunoblots (of $n > 3$ independent experiments) in U2OS cells, β-actin or β-tubulin were used as a loading controls. **a**, Cells were pre-treated with 10 μM ATM inhibitor, 2.5 μM ATR inhibitor, 2 μM DNAPK inhibitor or DMSO, irradiated (6 Gy) or left untreated, and harvested at indicated time points. **b**, H2AX levels of cells treated with siH2AX or siControl for 24 h. **c**, MDC1 levels in cells treated with siMDC1 or siControl for 24 h and 48 h. **d**, PFKFB3 levels in cells treated with siPFKFB3#1 and siPFKFB3#2 or siControl for 24 h and 48 h. **e**, RAD51 levels of U2OS cells treated with siRAD51 or siControl for 24 h and 48 h. **f**, RRM2 levels of U2OS cells treated with siRRM2 or siControl for 24 h.

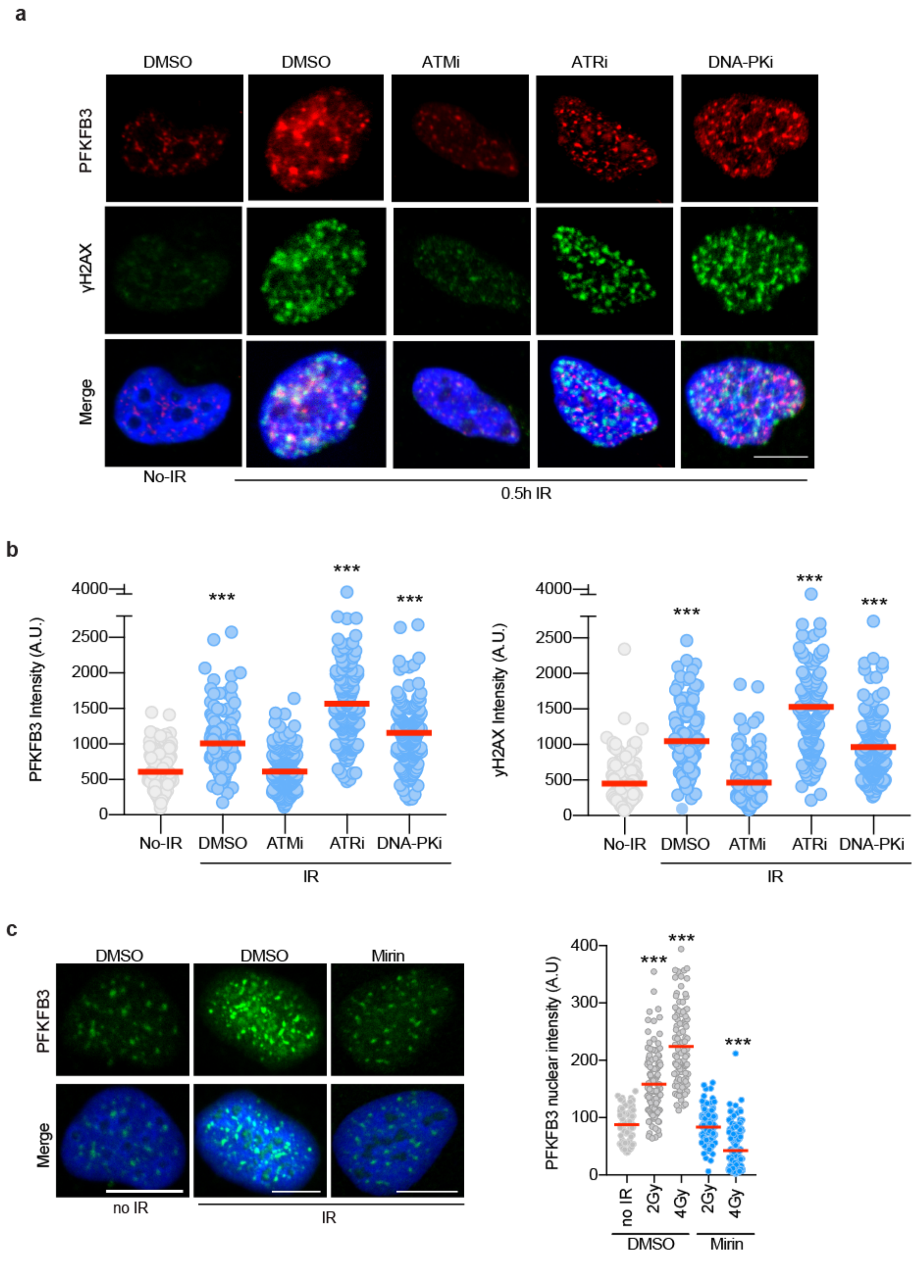

Supplementary Figure 3. PFKFB3 is recruited by ATM and the MRN complex upon IR. a, Representative confocal images (of *n* > 2) of yH2AX and PFKFB3 nuclear recruitment after *in situ* fractionation in BJ RAS cells upon 6 h treatment with indicated inhibitor, exposed to IR (6 Gy, 0.5 h recovery) or left untreated. Scale bar, 10 μm. **b,** Scatter plot representing PFKFB3 (left) and yH2AX (right) nuclear intensity in (**a**) quantified using CellProfiler, > 100 cells / condition. *** *P*<0.001. **c,** Confocal analysis of nuclear PFKFB3 in U2OS cells treated with 500μM Mirin inhibitor for 2 h or DMSO followed by IR (6 Gy, 2 h recovery), or left untreated. Scale bar, 10 μm. To the right, scatter plot showing PFKFB3 nuclear intensity in U2OS cells pre-treated with 500μM Mirin inhibitor for 2 h or DMSO and then irradiated at indicated Gy. Cells were fixed 2 h post-irradiation, >100 cells/condition (*n* = 2 independent experiments). Scale bar, 10 μm. *** *P*<0.001. One-way ANOVA was used to calculate statistical significance.

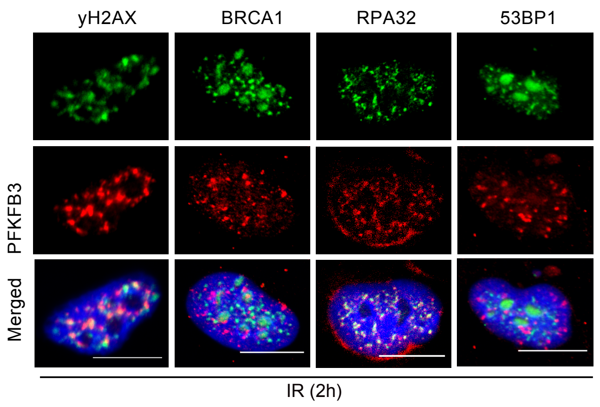

Supplementary Figure 4. Co-localization of PFKFB3 and HR factors. Representative confocal images of recruitment and co-localization of PFKFB foci with HR factors foci in IR-treated BJ RAS cells (6Gy, 2 h recovery). *n* = 2 independent experiments. Scale bar, 10 μm.

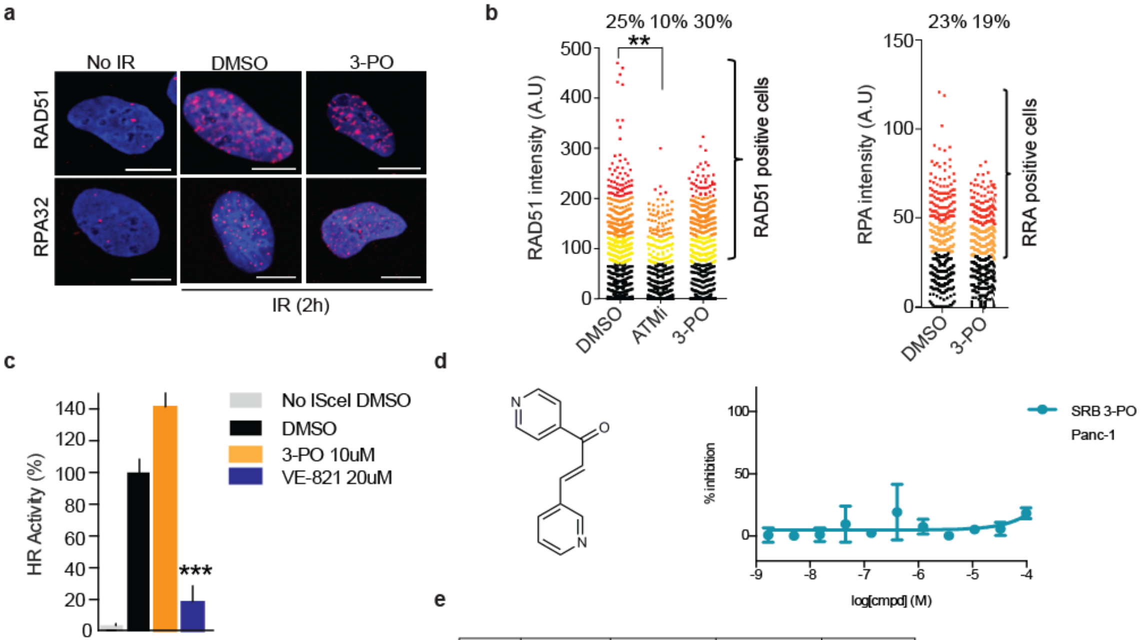

e

	PFKFB3 Activity	Panc-1/vS assay (uM)	Panc-1/SRB assay (uM)	SW620/SRB assay (uM)
3-PO	No IC50	No IC50	16.3	12.2

Supplementary Figure 5. Effects of 3-PO compound on DNA repair and PFKFB3 inhibition. a, Confocal analysis of IR-induced foci of RAD51 or RPA32 in U2OS cells following 6 h treatment with DMSO or 10 µM 3-PO, exposed to IR (6Gy, 2h recovery) or left untreated. Scale bar, 10 µm. **b**, Scatter dot plot representing the intensity of RAD51 or RPA32 in (**a**) as assessed by quantifying nuclear intensity using CellProfiler, n > 500 cells/treatment (n = 3 independent experiments). ANOVA was used to calculate statistical significance, ** P<0.01. **c**, HR activity of U2OS DR-GFP cells treated with indicated inhibitors as assessed by FACS analysis. DMSO treated cells are taken as reference cells (100% activity), data are displayed as means ± SEM, n=3. One-way ANOVA was used to calculate statistical significance, *** P<0.001. **d**, Chemical structure of 3-PO inhibitor. To the right, inhibition of recombinant human PFKFB3 by increasing concentrations of 3-PO as assessed by the ADP-GLO assay. Data are displayed as mean ± SD, n = 2. **e,** Summary of 3-PO inhibition of human PFKFB3. First column shows IC_{50} value of 3-PO using the ADP-GLO assay, second column indicates IC_{50} value for the formation of F-2,6-P_2 in PANC-1 cells using the biochemical van Schaftingen assay. Last two columns indicate IC_{50} values of cell viability assays in PANC-1 and SW620 cells using the SRB assay, n = 2.

a

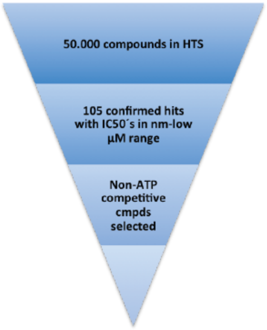

b

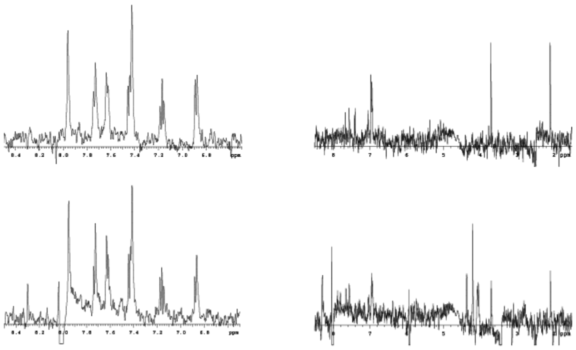

c

	IC50 (µM)	+/- STD	N
PFKFB1	ND		1
PFKFB2	ND		1
PFKFB3	0.19	0.04	5
PFKFB4	3.30		1

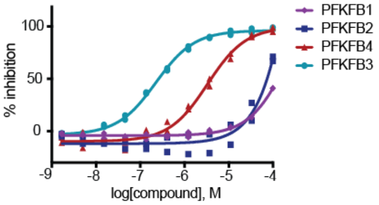

d

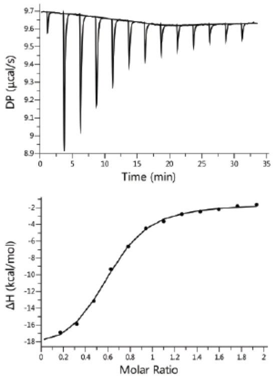

e

	IC50 (µM)	+/- STD	N
Mia Paca-2	0.24	0.04	3
NUGC3	0.09	0.02	2
PANC1	0.19	0.07	5
SW620	0.60	0.27	4

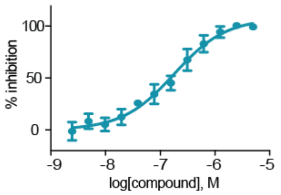

f

	IC50 (µM)	+/- STD	N
MiaPaca-2	2.75	0.49	2
PANC1	3.83	0.93	2
SW620	7.50	0.00	2
PBMC	N.D.		8
U-266	5.08	3.03	11
AMO-1	11.53	4.63	4

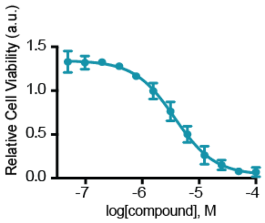

g

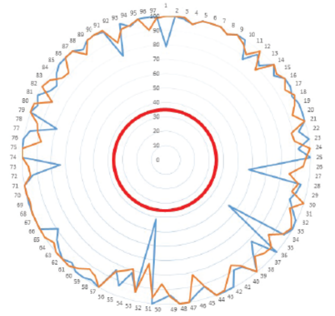

Supplementary Figure 6. PFKFB3 inhibitor screening and optimization.
a, 50,000 small molecules were screened for inhibition of human recombinant PFKFB3 using the EasyLite assay. Non-ATP competitive ligands were selected for further optimization to avoid cross activity with other kinases. **b**, 1H Saturation Transfer Difference NMR spectra of samples containing PFKFB3 and KAN0104821 (left panel) or KAN0082924 (right panel). Upper and lower panel, respectively ,represent conditions without or with large excess of ATP. The compound signals in the top spectrums show that both compounds bind reversibly to PFKFB3. For KAN0104821 the compound signal intensities do not decrease in the presence of a large excess of ATP showing that it does not compete with ATP, whereas for KAN0082924 a significant decrease is seen showing that the compound competes for binding with ATP. **c**, Inhibition of the four isoenzymes PFKFB1-4 by KAN0438241 as assessed by the ADP-GLO assay. The table holds mean IC_{50} values ± STD. To the right, representative dose-response curves for each isoenzyme is shown, *n = 2*. **d**, Isothermal titration calorimetry (ITC) assay for titration of KAN0438241 into PKFBF3 protein. Top image shows raw ITC and the bottom image shows the binding isotherm curve with integrated heats of injections overlaid on the best fit to one-set-of-sites binding model. Data analysis was performed with dedicated PEAQ-ITC analysis software. **e**, Measurement of intracellular F-2,6-P_2 levels (0.25 % serum) upon KAN0438757 treatment using the van Schaftingen assay. The table holds mean IC_{50} ± SD. To the right, representative curve of intracellular F-2,6-P_2 levels in Panc-1 cells, *n* = 5 independent experiments. **f**, Cell viability (5 % serum) in indicated cell lines treated with KAN0438757 for 72 h assessed by two methods: the SRB assay and CellTiterGlo. The table holds mean IC_{50} ± SD. To the right, representative cell viability curve in PANC-1 using the SRB method, *n* = 3 independent experiments. **g,** Polar circle diagram displaying selectivity profile of KAN0438757 (blue) and KAN0438241 (red) for 97 kinases in the DiscoveRx's diverse scanEDGE^{sm}/ KINOMEscan set of kinases. The red circle in the middle represents the cut-off value for significant binding. The number labels represent each kinase (see Supplementary Data information to identify each kinase). N = number of test occasions.

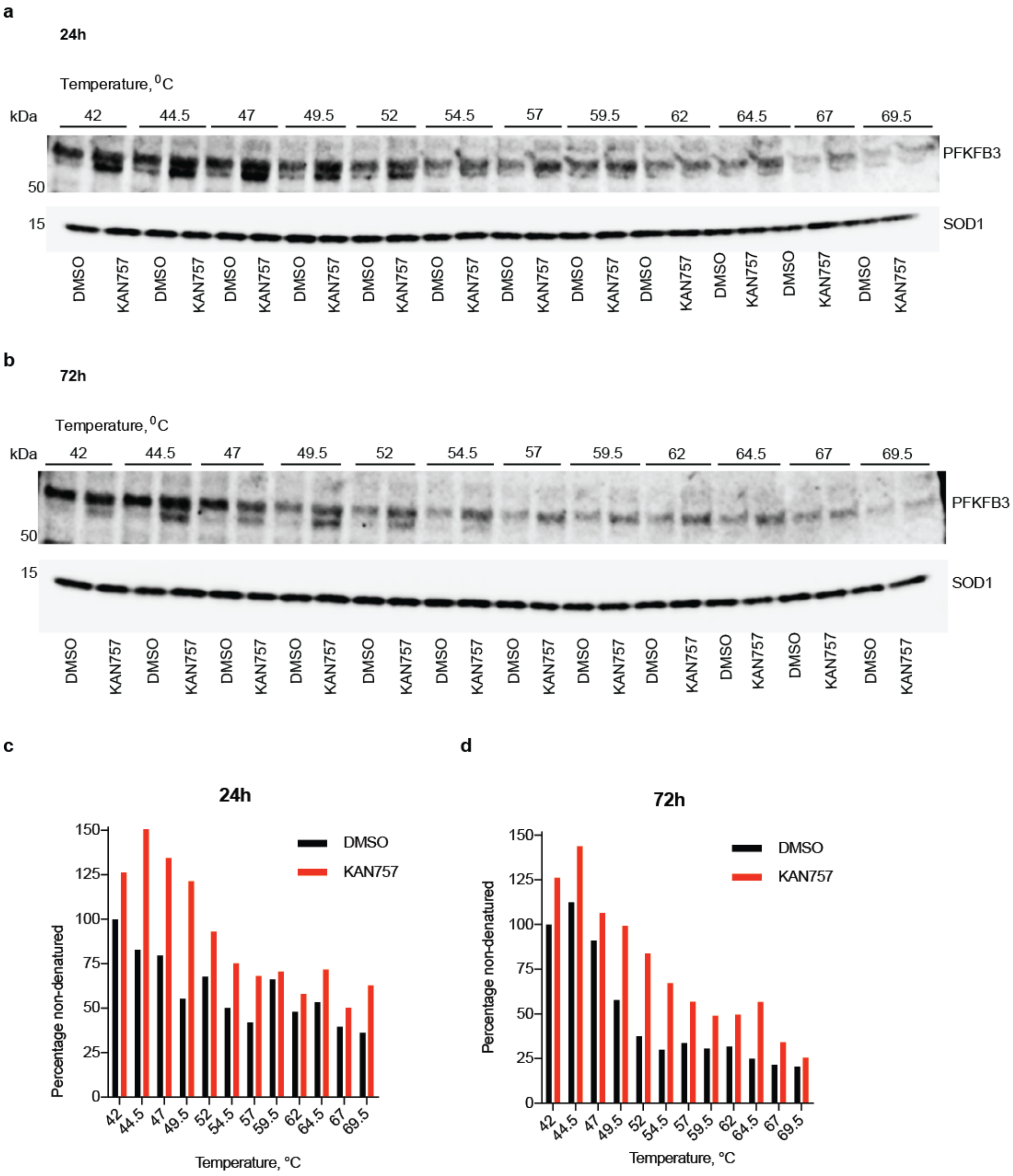

Supplementary Figure 7. Thermal shift of PFKFB3 with KAN0438757
a, Representative immunoblot (of n = 2) to assess target engagement and thermal stabilization of PFKFB3 by 10 μM KAN757 treatment for 24 h compared to DMSO in U2OS cells. **b**, Representative immunoblot (of n = 2) to assess target engagement and thermal stabilization of PFKFB3 by 10 μM KAN757 treatment for 72 h compared to DMSO in U2OS cells. **c**, Percentage of non-denatured PFKFB3 relative to DMSO in (**a**, 24h treatment). Densitometric quantification of band intensities was performed using Image J, PFKBF3 levels were normalized against SOD1. **d**, Percentage of non-denatured PFKFB3 relative to DMSO in (**b**, 72h treatment). Densitometric quantification of band intensities was performed using Image J, PFKBF3 levels were normalized against SOD1.

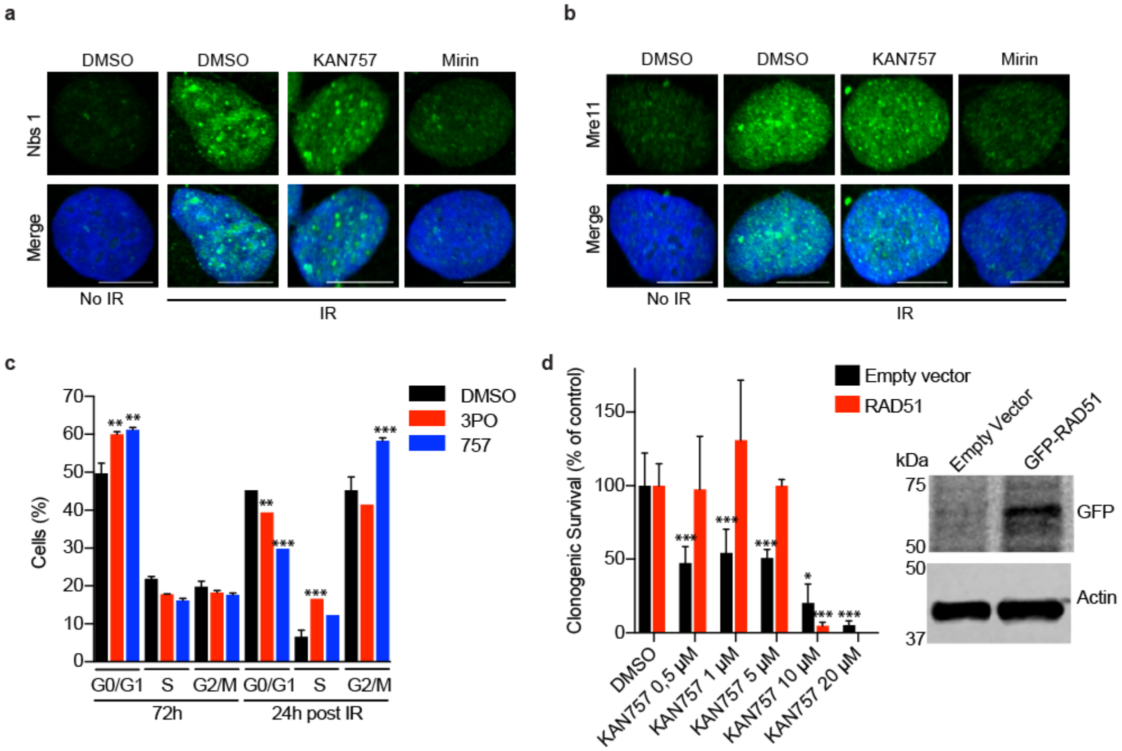

Supplementary Figure 8. PFKFB3 acts downstream of the MRN complex. a, Representative confocal images (of $n = 2$) of Nbs1 in U2OS cells pre-treated with 10 µM KAN757 (6 h) or 500µM Mirin (2 h), exposed to IR (6 Gy, 2 h recovery) or left untreated. Scale bar, 10 µm. **b**, Representative confocal images (of $n = 2$) of Mre11 in U2OS cells pre-treated with 10 µM KAN757 (6 h) or 500µM Mirin (2 h), exposed to IR (6 Gy, 2 h recovery) or left untreated. **c**, DNA histograms of U2OS cells treated with indicated inhibitors for 2 h following IR at 2 Gy (24 h recovery) or treated for 72 h (non-irradiated). Cells were fixed and DNA content was assessed using propidium iodide staining and flow cytometry. Data are displayed as mean +/- SEM, n=3. ** P<0.01, *** P<0.001; One-way ANOVA . **d**, Clonogenic survival of BJ RAS cells transfected with empty vector or GFP-RAD51 plasmid and treated with indicated concentrations of KAN757 or vehicle. Cells were exposed to IR (2Gy) and inhibitors washed out 72 h post IR. Colonies were fixed after 12 days and stained with 4 % methylene blue in MeOH. To the right, representative immunoblot of empty vector and GFP-RAD51 transfected cells to confirm overexpression of RAD51. Data are displayed over DMSO empty vector or DMSO GFP-RAD51 correspondingly, mean +/- SEM, n=3. * P<0.05, *** P<0.001. One-way ANOVA was used to calculate statistical significance.

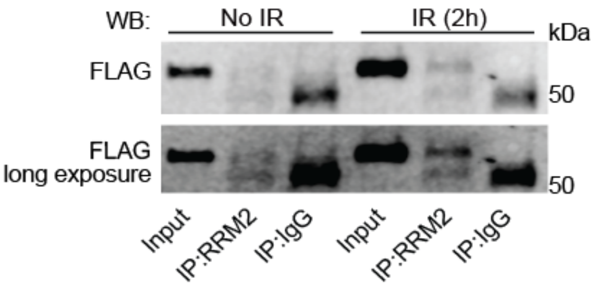

Supplementary Figure 9. PFKFB3 interacts with RRM2.
FLAG-PFKFB3 transfected U2OS cells were exposed to IR (6 Gy) or left untreated. After 2 h recovery, cells were collected and subjected to immunoprecipitation and immunoblot, *n* = 2. IgG, immunoglobulin.

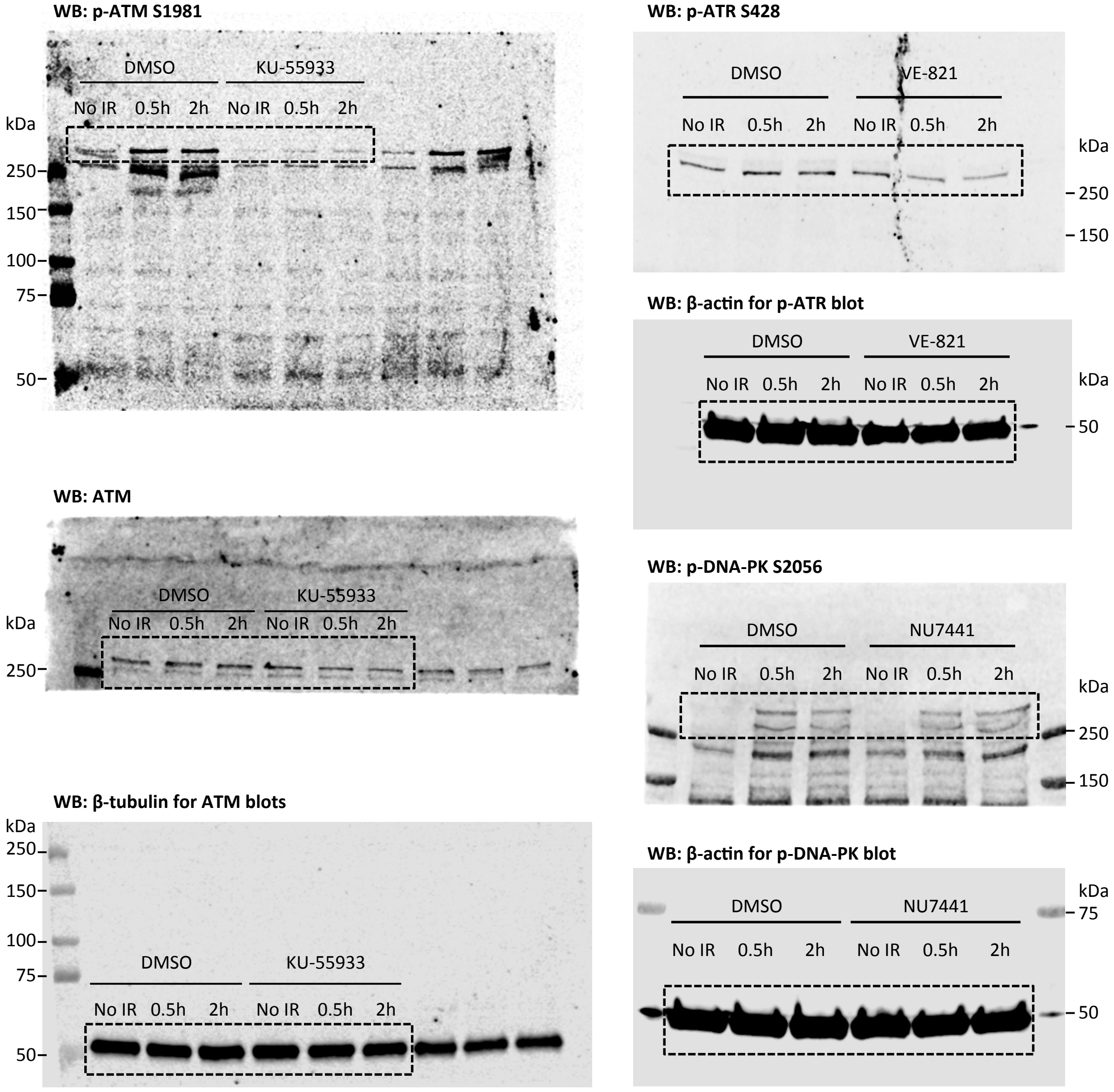

Supplementary Figure 10. Uncropped western blots for Supplementary Figure 2a. Dotted lines indicate cropped area.

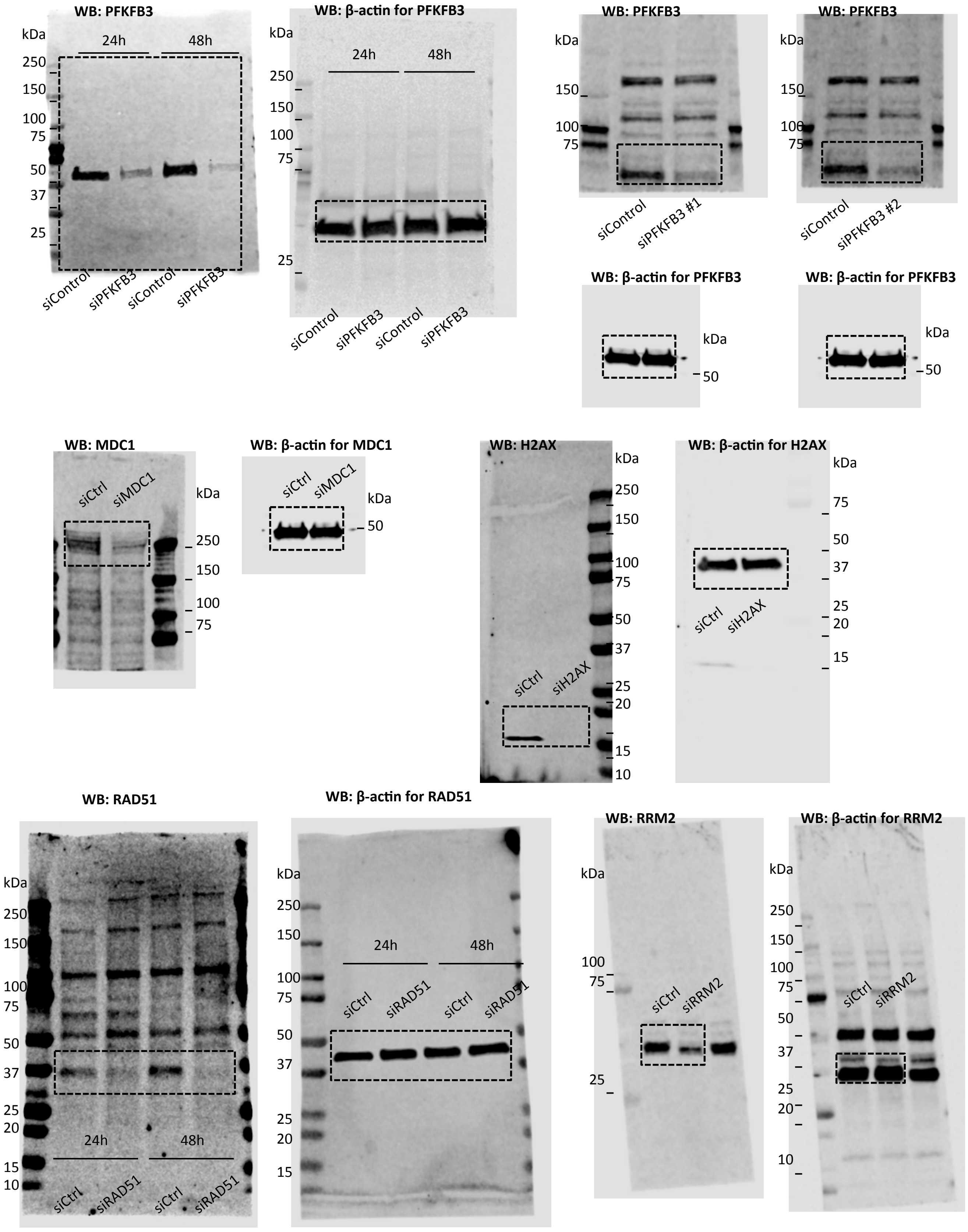

Supplementary Figure 11. Uncropped western blots for Supplementary Figure 2b-d. Dotted lines indicate cropped area.

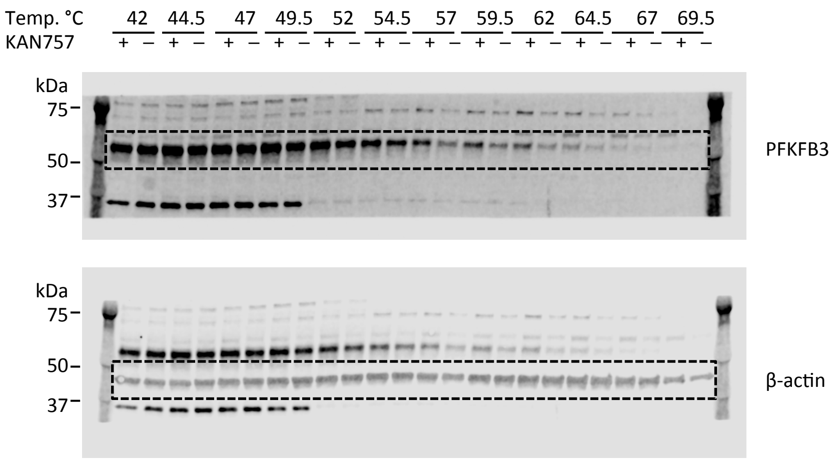

Supplementary Figure 12. Uncropped western blots for Figure 3g. Dotted lines indicate cropped area.

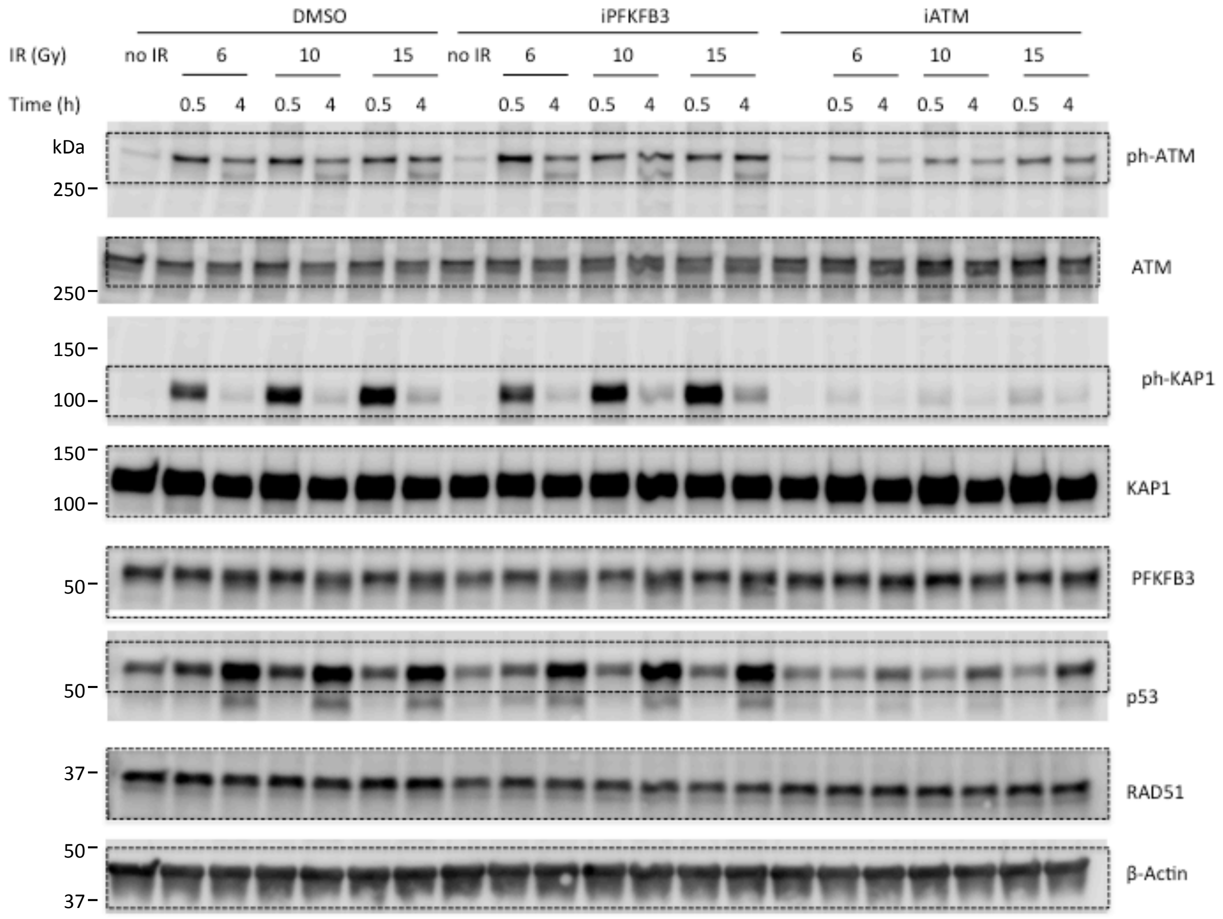

Supplementary Figure 13. Uncropped western blots for Figure 4e. Dotted lines indicate cropped area.

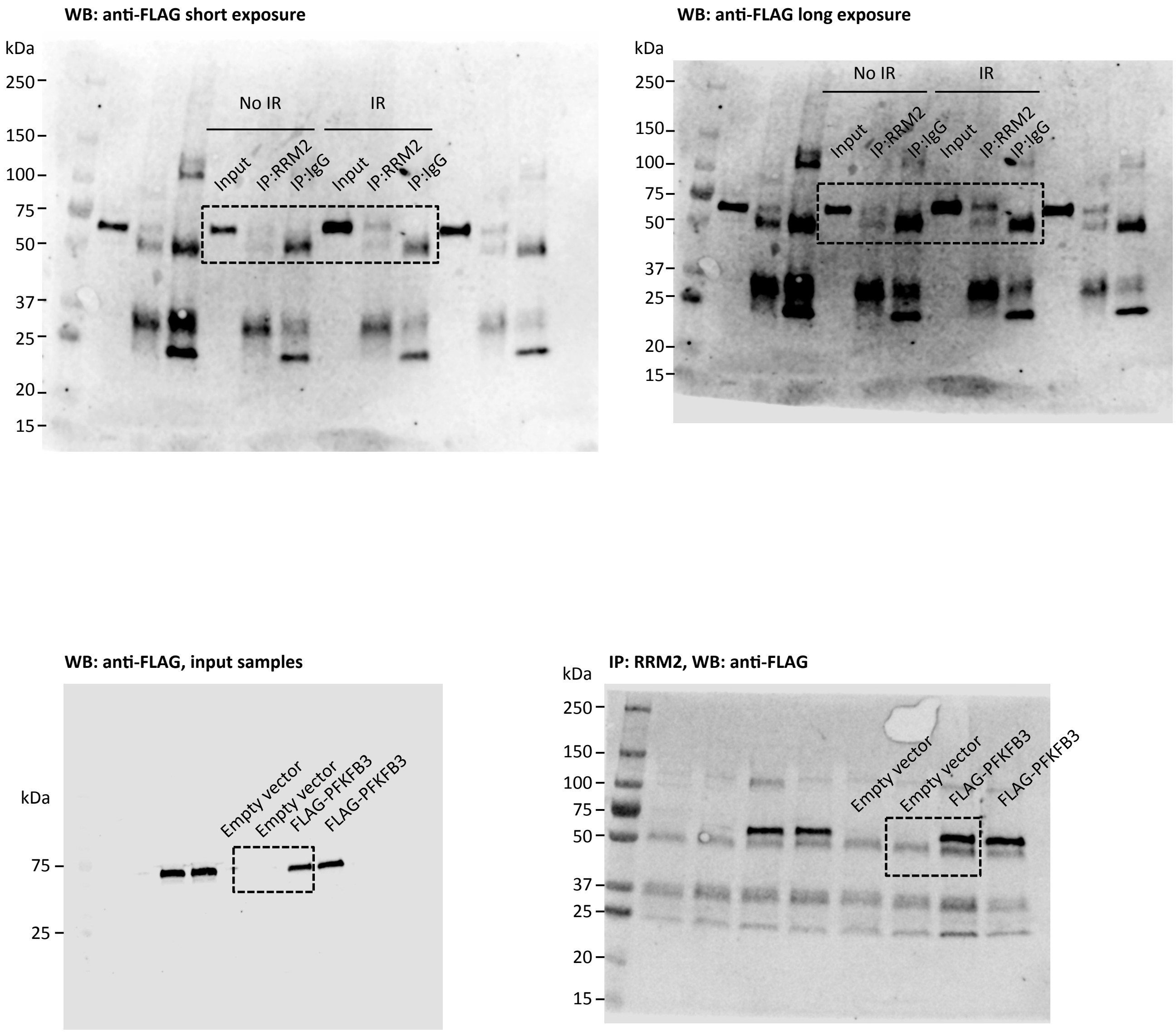

Supplementary Figure 14. Uncropped western blots for Figure 5d and Supplementary Figure 9. Dotted lines indicate cropped area.

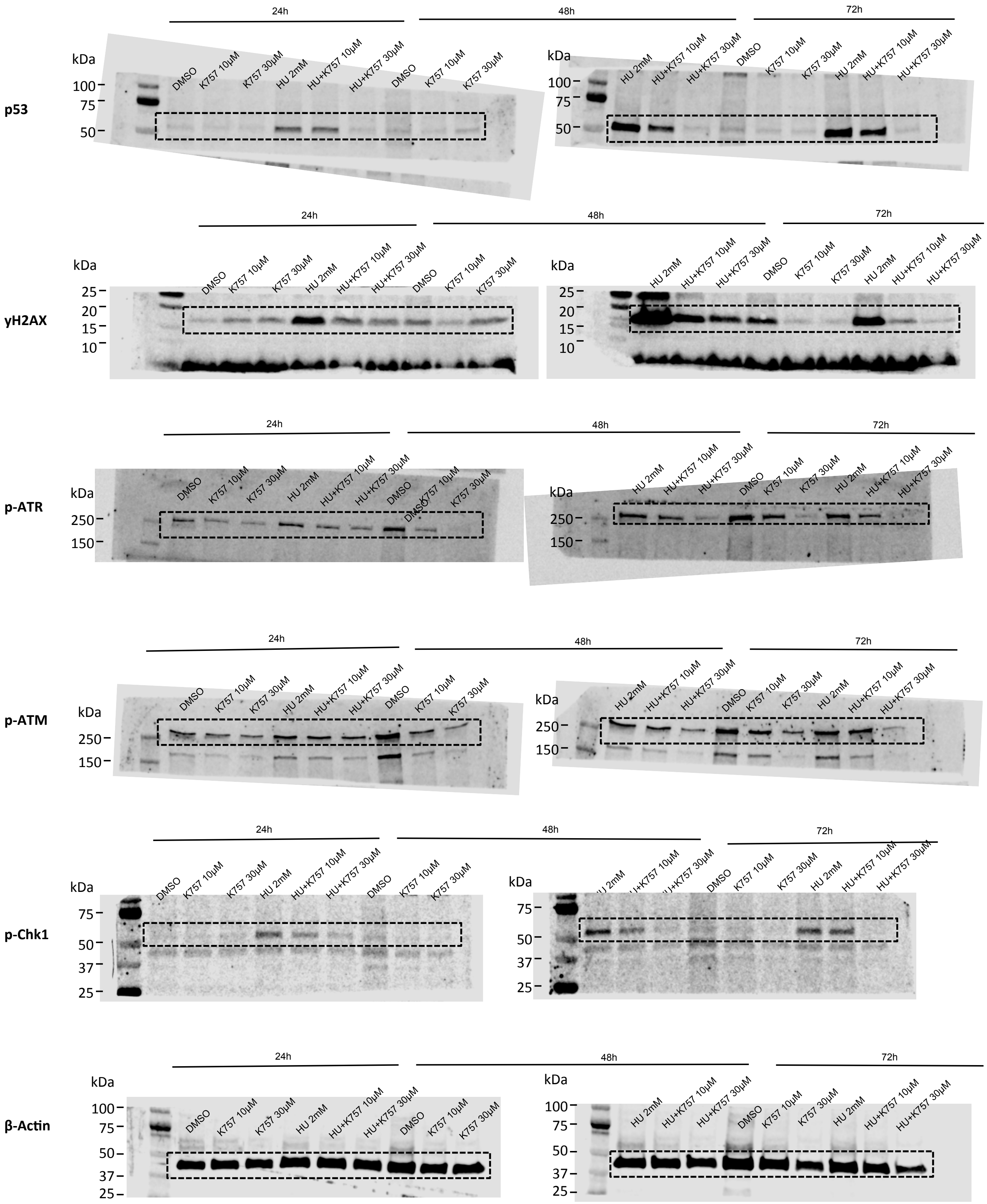

Supplementary Figure 15. Uncropped western blots for Figure 6f. Dotted lines indicate cropped area.

24h

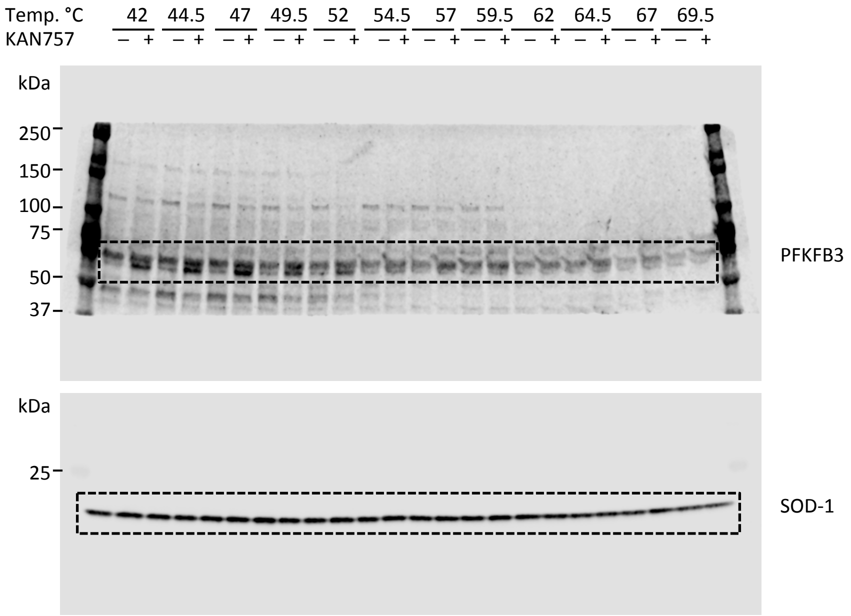

72h

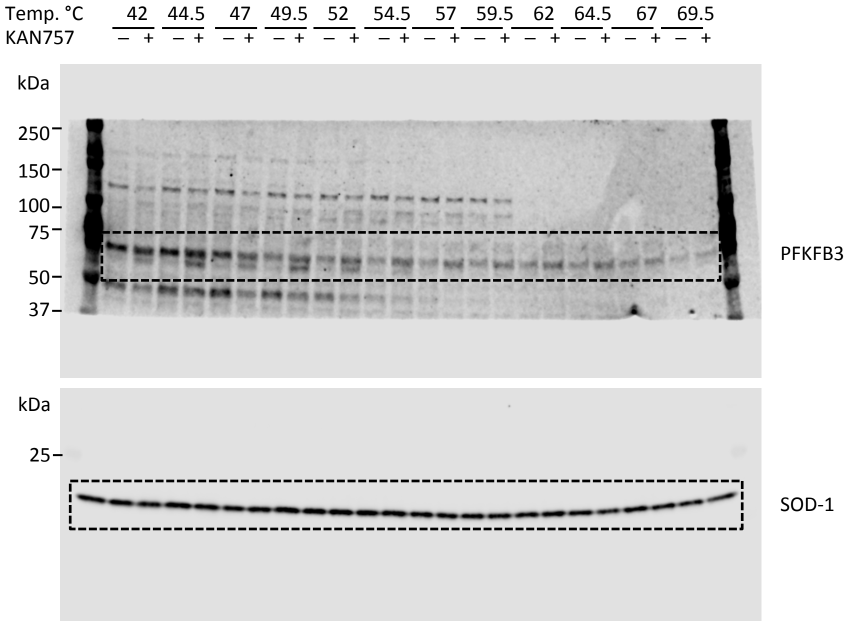

Supplementary Figure 16. Uncropped western blots for Supplementary Figure 7. Dotted lines indicate cropped area.
