# Supplementary material for: Targeting PFKFB3 radiosensitizes cancer cells and suppresses homologous recombination
Source: Nat Commun. 2018 Sep 24;9:3872. doi: 10.1038/s41467-018-06287-x (PMC6155239; doi:10.1038/s41467-018-06287-x)
Supplement: Supplementary file 2 — Description of Additional Supplementary Files [file 41467_2018_6287_MOESM2_ESM.docx]

**Description of Additional Supplementary Files**

File Name: Supplementary Data 1

Description: Selectivity profile of KAN0438757 and KAN0438241 for 97 kinases in the DiscoveRx’s diverse scanEDGE^sm^/KINOMEscan set of kinases. The number labels represent each kinase.
