# Supplementary material for: Targeting PFKFB3 radiosensitizes cancer cells and suppresses homologous recombination
Source: Nat Commun. 2018 Sep 24;9:3872. doi: 10.1038/s41467-018-06287-x (PMC6155239; doi:10.1038/s41467-018-06287-x)
Supplement: Supplementary file 3 — Supplementary Dataset 1 [file 41467_2018_6287_MOESM3_ESM.pdf]

Supplementary Data 1

Compound Name	DiscoveRx Gene Symbol	Entrez Gene Symbol	Percent Control	Compound Concentration (nM)
KAN0438241	ABL1(E255K)-phosphorylated	ABL1	100	2000
KAN0438241	ABL1(T315I)-phosphorylated	ABL1	100	2000
KAN0438241	ABL1-nonphosphorylated	ABL1	100	2000
KAN0438241	ABL1-phosphorylated	ABL1	96	2000
KAN0438241	ACVR1B	ACVR1B	100	2000
KAN0438241	ADCK3	CABC1	100	2000
KAN0438241	AKT1	AKT1	100	2000
KAN0438241	AKT2	AKT2	97	2000
KAN0438241	ALK	ALK	100	2000
KAN0438241	AURKA	AURKA	97	2000
KAN0438241	AURKB	AURKB	92	2000
KAN0438241	AXL	AXL	100	2000
KAN0438241	BMPR2	BMPR2	88	2000
KAN0438241	BRAF	BRAF	100	2000
KAN0438241	BRAF(V600E)	BRAF	97	2000
KAN0438241	BTK	BTK	100	2000
KAN0438241	CDK11	CDK19	96	2000
KAN0438241	CDK2	CDK2	100	2000
KAN0438241	CDK3	CDK3	97	2000
KAN0438241	CDK7	CDK7	93	2000
KAN0438241	CDK9	CDK9	100	2000
KAN0438241	CHEK1	CHEK1	89	2000
KAN0438241	CSF1R	CSF1R	90	2000
KAN0438241	CSNK1D	CSNK1D	100	2000
KAN0438241	CSNK1G2	CSNK1G2	97	2000
KAN0438241	DCAMKL1	DCLK1	95	2000
KAN0438241	DYRK1B	DYRK1B	86	2000
KAN0438241	EGFR	EGFR	93	2000
KAN0438241	EGFR(L858R)	EGFR	90	2000
KAN0438241	EPHA2	EPHA2	100	2000
KAN0438241	ERBB2	ERBB2	92	2000
KAN0438241	ERBB4	ERBB4	96	2000
KAN0438241	ERK1	MAPK3	99	2000
KAN0438241	FAK	PTK2	96	2000
KAN0438241	FGFR2	FGFR2	88	2000
KAN0438241	FGFR3	FGFR3	95	2000
KAN0438241	FLT3	FLT3	94	2000
KAN0438241	GSK3B	GSK3B	91	2000
KAN0438241	IGF1R	IGF1R	100	2000

KAN0438241	IKK-alpha	CHUK	90	2000
KAN0438241	IKK-beta	IKBKB	86	2000
KAN0438241	INSR	INSR	92	2000
KAN0438241	JAK2(JH1domain-catalytic)	JAK2	99	2000
KAN0438241	JAK3(JH1domain-catalytic)	JAK3	100	2000
KAN0438241	JNK1	MAPK8	91	2000
KAN0438241	JNK2	MAPK9	82	2000
KAN0438241	JNK3	MAPK10	100	2000
KAN0438241	KIT	KIT	100	2000
KAN0438241	KIT(D816V)	KIT	95	2000
KAN0438241	KIT(V559D,T670I)	KIT	86	2000
KAN0438241	LKB1	STK11	96	2000
KAN0438241	MAP3K4	MAP3K4	73	2000
KAN0438241	MAPKAPK2	MAPKAPK2	94	2000
KAN0438241	MARK3	MARK3	79	2000
KAN0438241	MEK1	MAP2K1	82	2000
KAN0438241	MEK2	MAP2K2	85	2000
KAN0438241	MET	MET	100	2000
KAN0438241	MKNK1	MKNK1	94	2000
KAN0438241	MKNK2	MKNK2	93	2000
KAN0438241	MLK1	MAP3K9	97	2000
KAN0438241	p38-alpha	MAPK14	100	2000
KAN0438241	p38-beta	MAPK11	100	2000
KAN0438241	PAK1	PAK1	100	2000
KAN0438241	PAK2	PAK2	97	2000
KAN0438241	PAK4	PAK4	100	2000
KAN0438241	PCTK1	CDK16	100	2000
KAN0438241	PDGFRA	PDGFRA	100	2000
KAN0438241	PDGFRB	PDGFRB	100	2000
KAN0438241	PDPK1	PDPK1	99	2000
KAN0438241	PIK3C2B	PIK3C2B	100	2000
KAN0438241	PIK3CA	PIK3CA	100	2000
KAN0438241	PIK3CG	PIK3CG	95	2000
KAN0438241	PIM1	PIM1	96	2000
KAN0438241	PIM2	PIM2	100	2000
KAN0438241	PIM3	PIM3	100	2000
KAN0438241	PKAC-alpha	PRKACA	88	2000
KAN0438241	PLK1	PLK1	91	2000
KAN0438241	PLK3	PLK3	93	2000
KAN0438241	PLK4	PLK4	100	2000
KAN0438241	PRKCE	PRKCE	100	2000
KAN0438241	RAF1	RAF1	89	2000
KAN0438241	RET	RET	100	2000
KAN0438241	RIOK2	RIOK2	98	2000

KAN0438241	ROCK2	ROCK2	92	2000
KAN0438241	RSK2(Kin.Dom.1-N-terminal)	RPS6KA3	91	2000
KAN0438241	SNARK	NUAK2	100	2000
KAN0438241	SRC	SRC	100	2000
KAN0438241	SRPK3	SRPK3	92	2000
KAN0438241	TGFBR1	TGFBR1	100	2000
KAN0438241	TIE2	TEK	100	2000
KAN0438241	TRKA	NTRK1	100	2000
KAN0438241	TSSK1B	TSSK1B	88	2000
KAN0438241	TYK2(JH1domain-catalytic)	TYK2	96	2000
KAN0438241	ULK2	ULK2	97	2000
KAN0438241	VEGFR2	KDR	100	2000
KAN0438241	YANK3	STK32C	91	2000
KAN0438241	ZAP70	ZAP70	99	2000
KAN0438757	ABL1(E255K)-phosphorylated	ABL1	79	2000
KAN0438757	ABL1(T315I)-phosphorylated	ABL1	100	2000
KAN0438757	ABL1-nonphosphorylated	ABL1	99	2000
KAN0438757	ABL1-phosphorylated	ABL1	96	2000
KAN0438757	ACVR1B	ACVR1B	100	2000
KAN0438757	ADCK3	CABC1	100	2000
KAN0438757	AKT1	AKT1	100	2000
KAN0438757	AKT2	AKT2	97	2000
KAN0438757	ALK	ALK	100	2000
KAN0438757	AURKA	AURKA	100	2000
KAN0438757	AURKB	AURKB	88	2000
KAN0438757	AXL	AXL	97	2000
KAN0438757	BMPR2	BMPR2	92	2000
KAN0438757	BRAF	BRAF	100	2000
KAN0438757	BRAF(V600E)	BRAF	95	2000
KAN0438757	BTK	BTK	100	2000
KAN0438757	CDK11	CDK19	100	2000
KAN0438757	CDK2	CDK2	98	2000
KAN0438757	CDK3	CDK3	100	2000
KAN0438757	CDK7	CDK7	100	2000
KAN0438757	CDK9	CDK9	100	2000
KAN0438757	CHEK1	CHEK1	94	2000
KAN0438757	CSF1R	CSF1R	100	2000
KAN0438757	CSNK1D	CSNK1D	92	2000
KAN0438757	CSNK1G2	CSNK1G2	100	2000
KAN0438757	DCAMKL1	DCLK1	74	2000
KAN0438757	DYRK1B	DYRK1B	58	2000
KAN0438757	EGFR	EGFR	95	2000
KAN0438757	EGFR(L858R)	EGFR	93	2000
KAN0438757	EPHA2	EPHA2	90	2000

KAN0438757	ERBB2	ERBB2	94	2000
KAN0438757	ERBB4	ERBB4	99	2000
KAN0438757	ERK1	MAPK3	100	2000
KAN0438757	FAK	PTK2	97	2000
KAN0438757	FGFR2	FGFR2	54	2000
KAN0438757	FGFR3	FGFR3	100	2000
KAN0438757	FLT3	FLT3	82	2000
KAN0438757	GSK3B	GSK3B	89	2000
KAN0438757	IGF1R	IGF1R	100	2000
KAN0438757	IKK-alpha	CHUK	91	2000
KAN0438757	IKK-beta	IKBKB	90	2000
KAN0438757	INSR	INSR	100	2000
KAN0438757	JAK2(JH1domain-catalytic)	JAK2	100	2000
KAN0438757	JAK3(JH1domain-catalytic)	JAK3	100	2000
KAN0438757	JNK1	MAPK8	92	2000
KAN0438757	JNK2	MAPK9	95	2000
KAN0438757	JNK3	MAPK10	100	2000
KAN0438757	KIT	KIT	100	2000
KAN0438757	KIT(D816V)	KIT	94	2000
KAN0438757	KIT(V559D,T670I),KIT	KIT	97	2000
KAN0438757	LKB1	STK11	100	2000
KAN0438757	MAP3K4	MAP3K4	42	2000
KAN0438757	MAPKAPK2	MAPKAPK2	94	2000
KAN0438757	MARK3	MARK3	82	2000
KAN0438757	MEK1	MAP2K1	93	2000
KAN0438757	MEK2	MAP2K2	84	2000
KAN0438757	MET	MET	100	2000
KAN0438757	MKNK1	MKNK1	98	2000
KAN0438757	MKNK2	MKNK2	100	2000
KAN0438757	MLK1	MAP3K9	100	2000
KAN0438757	p38-alpha	MAPK14	97	2000
KAN0438757	p38-beta	MAPK11	100	2000
KAN0438757	PAK1	PAK1	97	2000
KAN0438757	PAK2	PAK2	94	2000
KAN0438757	PAK4	PAK4	89	2000
KAN0438757	PCTK1	CDK16	100	2000
KAN0438757	PDGFRA	PDGFRA	100	2000
KAN0438757	PDGFRB	PDGFRB	100	2000
KAN0438757	PDPK1	PDPK1	100	2000
KAN0438757	PIK3C2B	PIK3C2B	100	2000
KAN0438757	PIK3CA	PIK3CA	100	2000
KAN0438757	PIK3CG	PIK3CG	93	2000
KAN0438757	PIM1	PIM1	74	2000
KAN0438757	PIM2	PIM2	100	2000

KAN0438757	PIM3	PIM3	100	2000
KAN0438757	PKAC-alpha	PRKACA	100	2000
KAN0438757	PLK1	PLK1	97	2000
KAN0438757	PLK3	PLK3	79	2000
KAN0438757	PLK4	PLK4	100	2000
KAN0438757	PRKCE	PRKCE	97	2000
KAN0438757	RAF1	RAF1	100	2000
KAN0438757	RET	RET	94	2000
KAN0438757	RIOK2	RIOK2	92	2000
KAN0438757	ROCK2	ROCK2	97	2000
KAN0438757	RSK2(Kin.Dom.1-N-terminal)	RPS6KA3	100	2000
KAN0438757	SNARK	NUAK2	100	2000
KAN0438757	SRC	SRC	100	2000
KAN0438757	SRPK3	SRPK3	96	2000
KAN0438757	TGFBR1	TGFBR1	100	2000
KAN0438757	TIE2	TEK	100	2000
KAN0438757	TRKA	NTRK1	99	2000
KAN0438757	TSSK1B	TSSK1B	78	2000
KAN0438757	TYK2(JH1domain-catalytic)	TYK2	100	2000
KAN0438757	ULK2	ULK2	96	2000
KAN0438757	VEGFR2	KDR	100	2000
KAN0438757	YANK3	STK32C	100	2000
KAN0438757	ZAP70	ZAP70	100	2000
